# Egr1 regulates the coordinated expression of numerous EGF receptor target genes as identified by ChIP-on-chip

**DOI:** 10.1186/gb-2008-9-11-r166

**Published:** 2008-11-25

**Authors:** Shilpi Arora, Yipeng Wang, Zhenyu Jia, Saynur Vardar-Sengul, Ayla Munawar, Kutbuddin S Doctor, Michael Birrer, Michael McClelland, Eileen Adamson, Dan Mercola

**Affiliations:** 1 Department of Pathology and Laboratory Medicine, University of California, Irvine, CA 92697, USA; 2 Sidney Kimmel Cancer Center, San Diego, CA 92121, USA; 3 Department of Periodontology, School of Dentistry, Ege University, Bornova 35100, Izmir, Turkey; 4 Burnham Institute for Medical Research, La Jolla, CA 92037, USA; 5 National Institutes of Health; Cell and Cancer Biology Branch, National Cancer Institute, Bethesda, MD 20892, USA

## Abstract

**Background:**

UV irradiation activates the epidermal growth factor receptor, induces Egr1 expression and promotes apoptosis in a variety of cell types. We examined the hypothesis that Egr1 regulates genes that mediate this process by use of a chip-on-chip protocol in human tumorigenic prostate M12 cells.

**Results:**

UV irradiation led to significant binding of 288 gene promoters by Egr1. A major functional subgroup consisted of apoptosis related genes. The largest subgroup of 24 genes belongs to the epidermal growth factor receptor-signal transduction pathway. Egr1 promoter binding had a significant impact on gene expression of target genes. Conventional chromatin immunoprecipitation and quantitative real time PCR were used to validate promoter binding and expression changes. Small interfering RNA experiments were used to demonstrate the specific role of Egr1 in gene regulation. UV stimulation promotes growth arrest and apoptosis of M12 cells and our data clearly show that a downstream target of the epidermal growth factor receptor, namely Egr1, mediates this apoptotic response. Our study also identified numerous previously unknown targets of Egr1. These include FasL, MAX and RRAS2, which may play a role in the apoptotic response/growth arrest.

**Conclusions:**

Our results indicate that M12 cells undergo Egr1-dependent apoptotic response upon UV stimulation and led to the identification of downstream targets of Egr1, which mediate epidermal growth factor receptor function.

## Abstract 2

UV stimulation of prostate cells causes an apoptotic response that is dependent on the zinc finger transcription factor Egr1; downstream targets of Egr1 in this response were identified.

## Background

Early growth response-1 (Egr1) is a zinc-finger nuclear phosphoprotein and transcription factor [1,2]. The gene for Egr1 (also known as Zif/268, NGFI-A and Knox24) encodes a 533 amino acid protein with 6 Cys_2_-His_2 _zinc finger motifs that exhibit partial homology to the gene sequence encoding the DNA binding domain of the Wilms tumor-1 suppressor (WT1) [3]. Indeed, both Egr1 and WT1 bind the Egr1 consensus regulatory sequence CGCCCCCGC in a zinc-dependent manner. Egr1 was first cloned as NGFI-A [4] from NGF-induced PC12 cells, and as Egr1 from mouse cells [1]. Early studies indicated its potential roles in cardiac and neural differentiation in a pluripotent EC (endothelial cells) line [1] and a role in monocytic differentiation of myeloid leukemia cells [5]. Subsequent studies have identified roles of Egr1 in cell growth, differentiation, inflammation associated with atherosclerosis [6], cardiac and pulmonary fibrosis [7,8] and a variety of roles in the physiology of the central nervous system.

In several types of human tumor cells, Egr1 exhibits suppressor gene activity via the binding to and transactivation of major tumor suppressor factors, including transforming growth factor-β1, p53, p73, and PTEN, indicating that Egr1 is a tumor suppressor factor (reviewed in [2]). Suppression of Egr1 expression is common in non-small cell lung cancers [9] and glioblastomas [10]. Egr1 is commonly deleted in the myelodysplastic syndrome (the acute myelogenous leukemia precursor condition), in mouse mutagenesis studies it induced myelodysplastic syndrome leukemogenesis, and acute myelogenous leukemia in mice was strongly associated with haploinsufficiency of Egr1 [11]. It has been proposed that Egr1 participates in, or coordinates a network of, tumor suppressor activities that serve to preserve contact inhibition of normal cells and promote anoikis of transformed variants [2].

In contrast, accumulating evidence based on *in vitro *studies, a survey of human surgical specimens, and transgenic mouse models indicate that Egr1 plays an important role in progression of prostate cancer [12]. Antisense Egr1 treatment of mouse prostate cell lines suppresses expression of Egr1 and several manifestations of transformation [13]. It has been suggested that Egr1 directly regulates genes that play a role in the development of prostate cancer [2,14]. A potential role of intracellular trafficking and posttranslational modification has also been implicated [15].

The expression of Egr1 is regulated in part through six CArG boxes located in the proximal 3' untranslated region of the Egr1 promoter [16]. CArG boxes, also known as serum response elements, have a consensus sequence CC(AT)_6_GG and bind phosphorylated serum response factor. The serum response factor is a major effector of the Map kinase/ERK pathway, mediator of a variety of growth factor receptors such as the epidermal growth factor receptor (EGFR) [17]. Activation of the EGFR leads to rapid expression of Egr1 in a variety of settings [18], including prostate cancer cells [19].

EGFR is strongly activated by a broad spectrum of irradiation [20]. The mechanism may involve the generation of reactive oxygen species [21] and may require the aggregation and internalization of EGFR [22]. Ultraviolet (UV) activation of EGFR is accompanied by the formation of complexes between activated EGFR and SOS (Son of sevenless), Grb2, phospholipase-Cγ (PLCγ) and SHC (Src homologous and collagen) [21]. Downstream signaling leads to rapid and transient activation of Egr1 expression. Activation is inhibited by suramin, suggesting that autocrine factors may mediate activation of the EGFR. UV stimulation later results in apoptosis. Here we examined human prostate M12 cells, a tumorigenic line derived from SV-40 immortalized P69 cells by serial passage in mice [23]. In these cells Egr1 is rapidly induced by treatment with UV radiation and serves as a model of Egr1 function. Our goal is to show that genes are bound by Egr1 in living cells upon UV stimulation, which provides a profile of genes more relevant to the mechanism of the EGFR pathway than expression analysis alone. We used a 'ChIP-on-chip' protocol and identified 288 promoters that were significantly bound by Egr1, which commonly functioned to regulate transcription. A large functionally related group of 24 genes is associated with the EGFR pathway and includes numerous mediators of apoptosis. Also, our results show several new targets of Egr1 (including MAX and RRAS2) that have previously not been associated with it. Indeed, UV treatment leads to inhibition of growth and apoptosis in an Egr1-dependent manner. The results illustrate that Egr1-regulated genes are required for the apoptotic response of UV treated prostate cancer cells.

## Results

### UV irradiation of M12 cells induces expression of endogenous Egr1 RNA and protein expression via the ERK1/2 pathway

**Figure 1:**
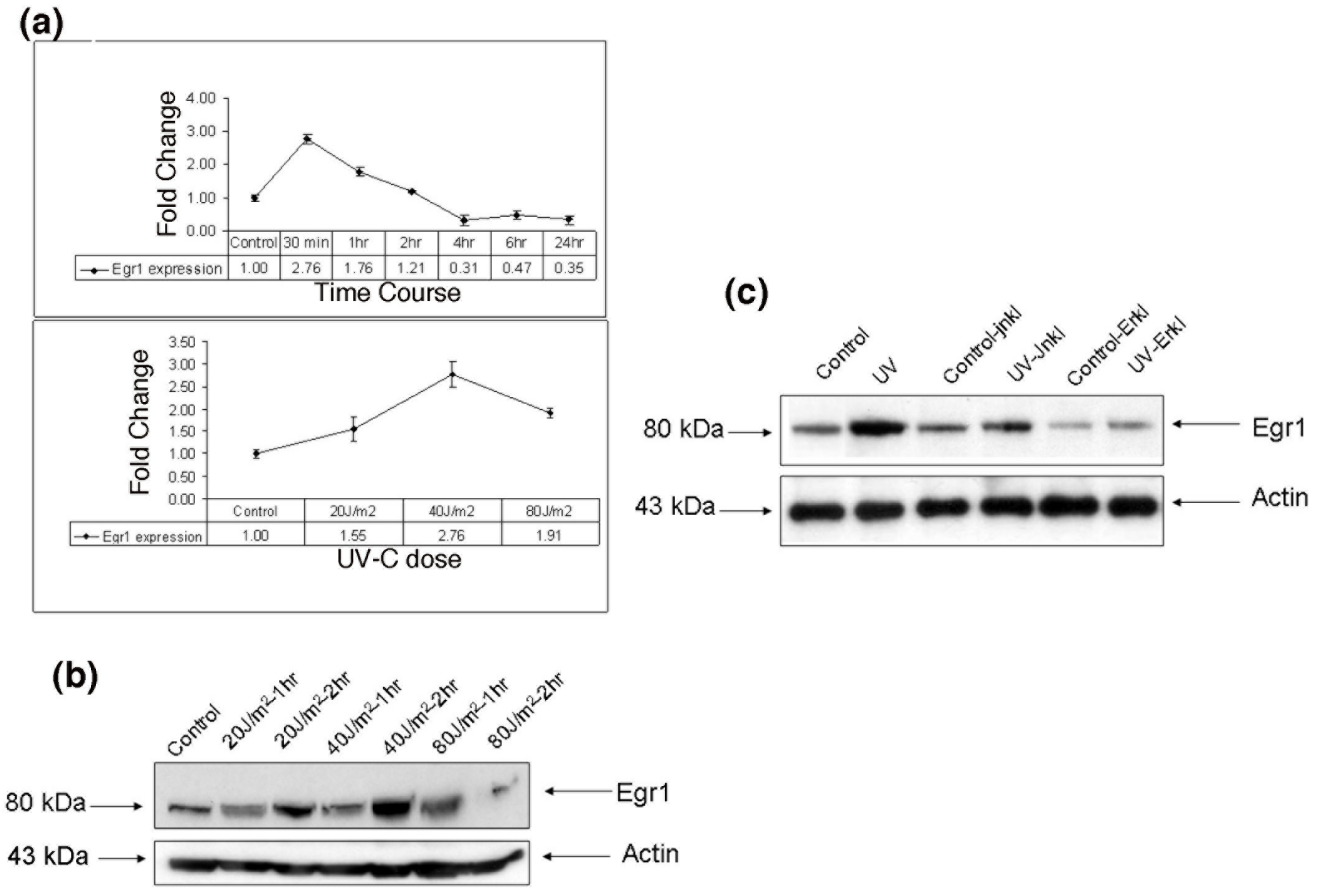
Egr1 induction upon UV-C stimulation. **(a) **Time course and dose response of Egr1 induction upon UV-C stimulation was measured by qRT-PCR. RNA was collected at various time points and at different doses of UV-C in order to determine the optimal dose (40 J/m^2^) and time point (2 h) for Egr1 expression. The upper panel shows the time course and the lower panel shows the dose response of Egr1 expression upon UV-C stimulation. **(b) **Western analysis using anti-Egr1 to define time of maximum UV response. **(c) **Western analysis of M12 cells treated with UV-C or not (control) in the presence and absence of ERK1/2 inhibitor (ErkI). The results show that prior treatment with ErkI resulted in negligible induction of Egr1 upon UV stimulation. Hence, at least 90% of Egr1 induction in these cells was downstream of the ERK MAP kinase.

Egr1 is barely detected in resting cells whereas irradiation with UV-C rapidly leads to markedly increased Egr1 expression. Dose-response and time-course experiments identified 40 J/m^2 ^as the optimal dose for Egr1 over-expression of mRNA and protein. Gene expression was increased approximately 3-fold at 30 minutes after treatment as measured by quantitative real time PCR (qRT-PCR; Figure 1a). Maximum protein expression was observed 2 h after UV irradiation (40 J/m^2^; Figure 1b). M12 cells are metastatic prostate cancer cells and we observed high basal expression of Egr1 in these cells compared to several other prostate cancer cell lines. We chose these cells, therefore, as our goal was to immunoprecipitate Egr1 from UV-treated cells and to use untreated cells as a true control for DNA immunoprecipitated from the UV-treated cells. We have shown earlier that stress stimuli, such as DNA-damaging agents that induce Egr1 expression, preferentially activate the stress activated Jun kinase pathway (JNK) and, to a lesser extent, the ERK1/2 pathway, while the p38 MAP kinase pathway is minimally affected in a variety of cell types [21,24]. To test whether ERK1/2 also may be involved in Egr1 expression following irradiation, M12 cells were treated with an ERK1/2 inhibitor, U0126, 45 minutes prior to UV stimulation. Egr1 expression remained at control levels in UV irradiated cells after treatment with U0126, whereas the cells that were treated with UV-C alone exhibited the characteristic induction of Egr1 protein, indicating that ERK1/2 acted upstream of Egr1 expression (Figure 1c). These results indicate that ERK1/2 is likely the dominant upstream MAP kinase pathway of induction of endogenous Egr1 protein expression in M12 cells.

### Chromatin immunoprecipitation revealed the formation of *in vivo* bound Egr1 DNA complexes

**Figure 2:**
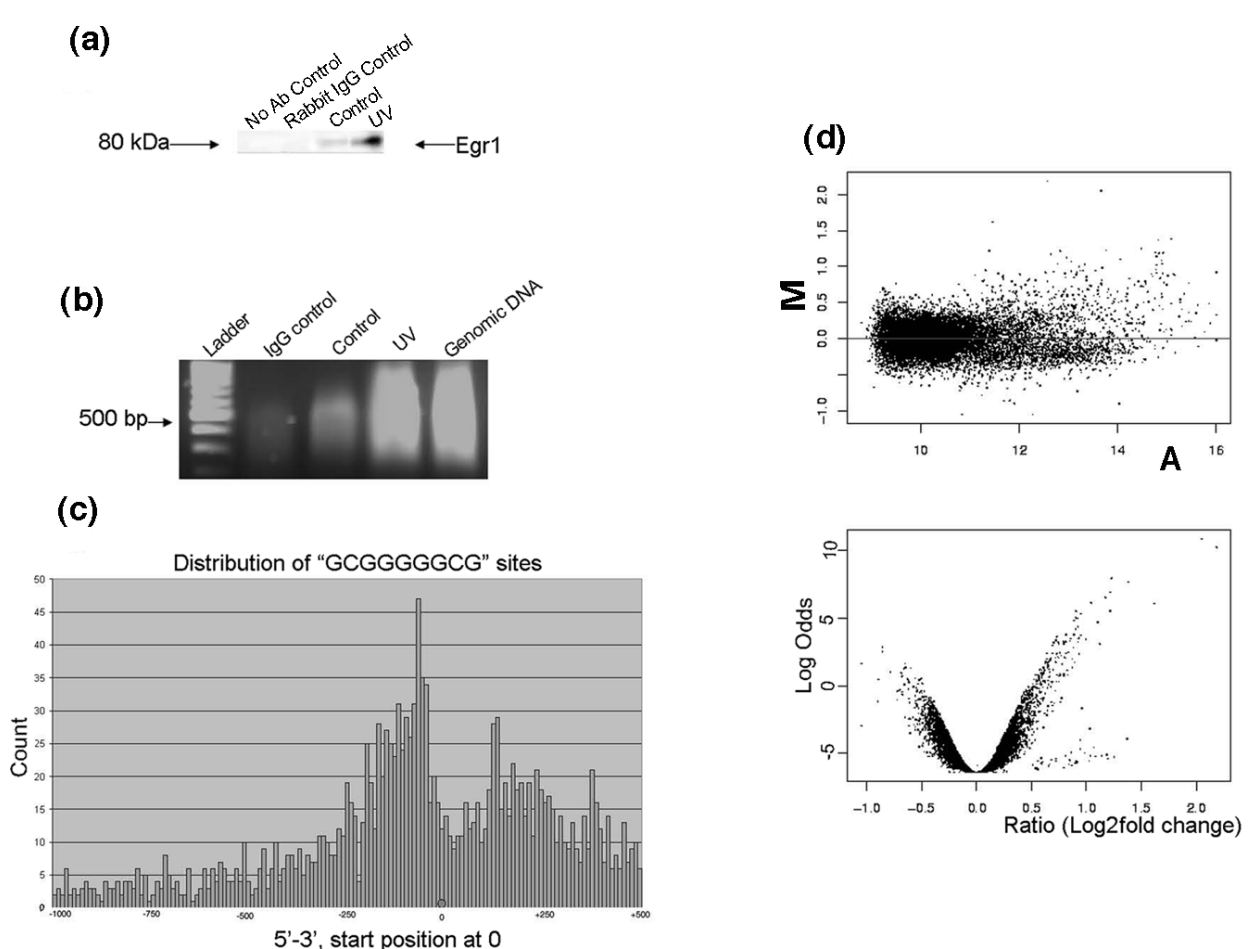
ChIP-on-chip hybridization results. **(a) **Western analysis of ChIP products for M12 cells treated with UV-C or not (control). **(b) **Yield and size of DNA precipitated with anti-Egr1 from M12 cells following treatment with UV-C. **(c) **Distribution of the consensus Egr1-binding sequence (5' GCGGGGGCG 3') in approximately 17,000 human genes. **(d) **M-A plots of promoter array hybridization intensities of ChIP products from control M12 cells and cells treated with 40 J/m^2 ^UV-C for 2 h. The lower panel shows significance (volcano) plots of the hybridization intensity data for ChIP products of M12 cells treated with UV-C for 2 h compared with control non-treated ChIP sample. The right arm of the lower plot shows significantly bound promoters.

To determine whether endogenous Egr1 protein of UV-stimulated cells was effectively translocated to the nucleus and bound DNA, we examined whether UV stimulation increased the binding of Egr1 to chromatin. Formaldehyde-crosslinked DNA was isolated from equal numbers of UV-stimulated and mock-stimulated cells, sonicated, and precipitated with anti-Egr1 (sc 110) antibody. Western analysis of anti-Egr1-precipitated DNA revealed Egr1, while Egr1 was barely detected in chromatin from control cells or chromatin pulled down with nonspecific IgG (Figure 2a). In addition, more DNA was recovered following UV irradiation compared to mock-treated cells. No detectable DNA was recovered from UV-treated cells when non-immune rabbit IgG control serum was used for chromatin immunoprecipitation (ChIP; Figure 2b). These results indicate that UV irradiation led to a large and specific increase in chromatin-bound Egr1.

### Identification of Egr1-bound promoters by promoter array hybridization

To identify the promoters bound by Egr1, we utilized promoter arrays containing approximately 12,000 promoter sequences amplified from normal human genomic DNA in the region of 500 nucleotides 3' of a known transcription start site to 1,000 nucleotides 5' of the transcription start site. This is the region of genes that contains many known functional transcriptional regulatory motifs, and is often the most CpG rich and G+C rich region in a gene [25]. Thus, this region is the most likely to harbor the CpG and G+C-rich consensus Egr1 binding site (EBS; 5' GCGGGGGCG 3'). A search for this motif in approximately 17,000 human genes with available annotation of transcription start sites in Refseq (NCBI reference sequences) revealed two major areas of Egr1 consensus binding motifs (Figure 2c). These regions were located at about -50 nucleotides 5' and about +100 nucleotides 3' of the transcription start site.

The ChIP-captured DNA from the UV-irradiated and non-irradiated cells were amplified in the presence of Cy3- or Cy5-conjugated nucleotide analogues, mixed in equal amounts and applied to the arrays. An M-A scatter plot of the combined data is shown in Figure 2d. The plot reveals a large population of increased array intensities (outliers) in the quadrant of positive M values and A > 11, indicating that UV stimulation preferentially leads to increased promoter binding by Egr1 in comparison to control DNA. Since the arrays are printed in triplicate, the experiment yields 12 array intensity measurements for each promoter. The fold changes are likely underestimates of the true change because the presence of any contaminating total genomic DNA in the ChIP samples reduces the dynamic range of the experiment. The significance (volcano) plots [26], which incorporate the B-values, confirm the existence of preferentially increased binding of DNA from UV-stimulated cells (Figure 2d). Array intensities judged as significantly increased were selected by two criteria: *p *< 0.005, and fold change >1.4. At least half of the genes also had a positive B value. The double criteria identified 288 gene promoters (5 were not considered further as they were cDNA clones with no sequence availability), which are listed in Table S1 in Additional data file 2. All the data files have been submitted to [GEO:GSE10585].

### Confirmation of the differential expression of UV-induced genes using bioinformatics criteria

Several observations indicate that the significant changes observed here accurately reflect differential precipitation and array binding. First, for the 283 genes that exhibited significantly altered hybridization following UV irradiation, 112/283 (39.6%) have perfect Egr1 consensus sites in their promoter sequences. Another 53 genes have probable EBSs (58.3% in total) whereas the frequency of EBSs in a set of 200 random sequences was only 23% (*p *< 0.005). Thus, the promoters reported as bound by Egr1 indeed contain a significant increase in the frequency of EBSs. Secondly, at least 43/283 (15%) genes are known to be UV responsive from other studies (Table S1 in Additional data file 2). A third indication comes from the identification of 24/283 significantly bound genes as EGFR-associated genes. These genes were identified by Pathway studio 5.0 (Ariadne Inc.; see Materials and methods), which compiles citations indicating that expression of these genes is associated with EGFR activity and/or expression. To evaluate this frequency, a set of 1,000 genes was examined in Pathway studio 5.0 using the same query, which yielded only 26 genes related to EGFR (*p *< 0.0001; Table S1 in Additional data file 2 and Figure S1 in Additional data file 4).

We examined the functional nature of the identified genes using program-assisted literature surveys such as Ariadne and Ingenuity (Materials and methods). Several functional groups of genes were apparent. These include regulators of apoptosis such as Bcl G, BLK, CASP7, BBC3 and also TNFSF5, TNFSF6 (FasL) and TNFSF19L, which belong to the tumor necrosis factor (TNF) family. Genes encoding the DNA repair enzymes NT5E, NME1 and NME2, cytokines, such as IL1R1, IL15 and IL18R1, the cell cycle regulators CDK8, CDKN1b/p27, PAK6 and SKP1a and the transcription regulators Ets2, Egr2, POU4F1, SOX11, EN1 and HSF4 were all among those containing significantly detected promoters. Genes such as *BBC3*, *PTPN13*, *MAX*, *MAP3K7 *and *MAP2K1 *(*MEK1*) and 38 others, have been previously documented as UV-responsive genes (Table S1 in Additional data file 2).

### Experimental validation of hybridization intensities

**Figure 3:**
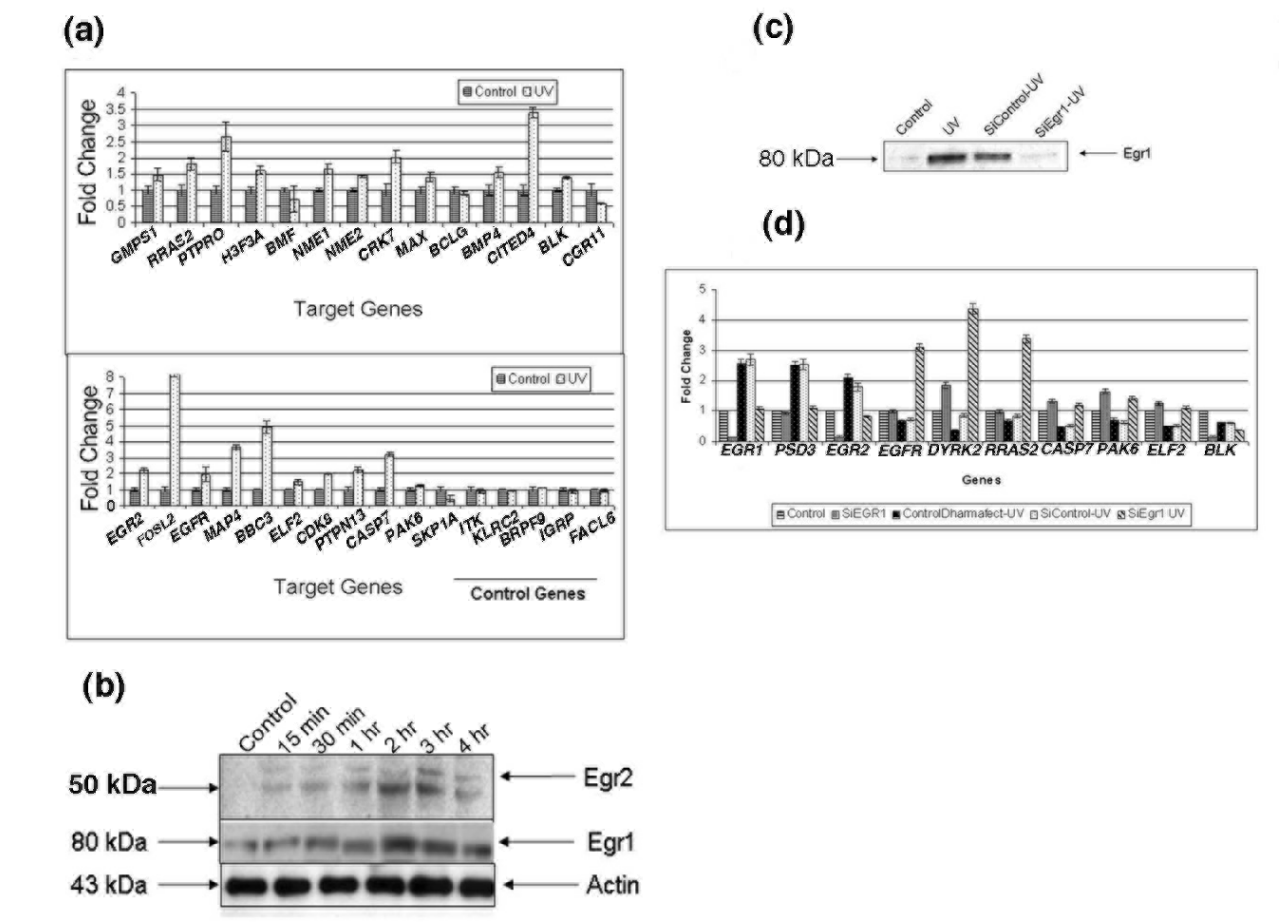
Validation of ChIP-on-chip results. **(a) **ChIP products for M12 cells treated with UV-C or untreated controls (control) were analyzed by qRT-PCR. The data are expressed as relative fold change of real-time PCR results to untreated control ChIP sample. The experiments were performed in triplicates and the error bars represent the triplicate data. **(b) **Western blot analysis of *Egr2 *(an Egr1 target gene) to define time of maximum induction. **(c) **M12 cells were treated with siRNA against Egr1 (SiEgr1) or SiGenome control (SiControl) for 48 h followed by UV-C irradiation and western blot confirmed the suppression of Egr1 in SiEgr1 treated cells. **(d) **qRT-PCR analysis of Egr1 target genes using RNA extracted from SiEgr1- or SiGenome control-treated M12 cells with or without UV-C irradiation. All the results are expressed relative to *GAPDH*.

Conventional ChIP was performed to confirm the results of 'ChIP-on-chip' experiments using a set of 25 representative genes. Primers were designed around the putative EBS on the target promoters and these were used for qRT-PCR amplification of the corresponding sequences from the ChIP-captured chromatin. The qRT-PCR results show that in 23/25 genes (92%), UV treatment led to increased PCR yields of 1.4- to 8-fold compared to control cells (Figure 3a). In contrast, little or no DNA enrichment was observed for all 25 primer sets when applied to precipitates prepared using control IgG serum. An additional five sets of primers for genes that were not on the 'significantly detected promoter' list and did not contain any EBS showed no DNA enrichment in the UV-stimulated samples. These observations indicate that the array intensities reliably reflect increased Egr1-DNA complex formation.

### Egr1 promoter binding regulates transcription

To determine whether Egr1 gene binding had an impact on transcription, Affymetrix gene expression analysis was carried out using U133plus2 arrays with approximately 54,000 probe sets. The analysis was performed on duplicate samples from M12 control and UV-irradiated cells. There were 2754 genes that showed significantly increased or decreased expression (2,754/54,675; 5%) as determined by the Affymetrix criteria (*GCOS v.1.4*). All the data files have been submitted to [GEO:GSE10585]. In order to determine whether the genes bound by Egr1 exhibit increased regulation and, therefore, potential phenotypic effects, we compared the average frequency of significant RNA changes of 5% with that observed for the 283 differentially bound promoters. This comparison revealed that twice as many genes (32/283, or 11.3%; Table S2 in Additional data file 3) exhibited significant changes in mRNA levels. The increased differential expression among the 283 Egr1 bound genes was significant (*p *< 0.001). Since numerous other non-Egr1 promoter-binding events potentially influence changes in transcription upon UV irradiation, only binding events that dominate regulation will be reflected in this analysis. It should be noted that binding events not associated with significant transcriptional change, either increased or decreased, do not provide evidence of false discovery of binding promoters nor evidence that Egr1 binding has no impact on transcription, but rather that the binding does not lead to a dominance over all other influences.

Thus, the result likely represents a minimum estimate of the regulatory influence of Egr1 binding. The result is further supported by comparison of the Affymetrix and qRT-PCR results. qRT-PCR was carried out on RNA for 37 genes chosen randomly from the 283 gene set. Of the 37 genes tested, 11 showed over-expression in UV-treated cells, while 21 had lower expression compared to the control cells (Table 1). Five genes did not show changes in gene expression. Genes with fold change values >1.5 were considered over-expressed, while ones that showed fold change values <0.5 in UV-treated cells compared to control cells were considered down-regulated. The levels of Egr2 were also verified at the protein level and there was concordance between the RNA and the protein levels demonstrating up-regulation of Egr2 (Figure 3b). Comparison of qRT-PCR with the Affymetrix data is limited as only 6 of these 37 chosen genes (16.2%) were among the significantly differentially expressed genes by the Affymetrix criteria. However, all values agreed in sign with an overall Pearson correlation coefficient of 0.784 (*p *= 0.06), indicating qualitative agreement between the Affymetrix intensity values and the qRT-PCR-measured expression changes. In a converse test, we compared the intensity values of all the 32 of the genes with significant Affymetrix expression changes to the corresponding M-values observed with the promoter arrays (Figure S2 in Additional data file 4). The genes exhibiting positive expression changes formed a well-resolved population characterized by a Pearson correlation coefficient of 0.68 (*p *= 0.001; Figure S2 in Additional data file 4).

**Table 1:** Gene expression analysis of Egr1 target genes using qRT-PCR of RNA extracted at various time points after UV-C treatment of M12 cells

	Fold change relative to control				
	
	30 minutes	1 h	2 h	4 h	6 h
*Egr2*	2.4	6.9	2.33	1.21	2.43
*PSD3*	17.2	25.3	9.40	4.86	1.05
*TOM22*	1.1	1.92	0.95	1.70	1.49
*H3A*	0.9	1.76	1.07	1.28	0.95
*BBC3*	1.6	0.71	0.23	0.29	2.28
*PTPRO*	1.1	0.20	0.44	0.31	20.47
*AKAP9*	1.4	1.26	1.59	0.66	1.00
*TNFSF6*	4.6	0.70	1.58	0.62	0.62
*CysLTR1*	2.07	2.28	3.39	0.66	2.81
*HK1*	1.43	1.28	1.72	0.91	1.10
*NME2*	0.82	1.35	2.05	1.53	1.13
*NME1*	1.06	1.21	1.15	1.08	1.29
*IFITM2*	0.70	0.88	0.66	0.95	0.77
*IL11RA*	0.78	0.98	0.77	0.86	0.80
*ABCC3*	0.79	1.06	0.71	0.70	0.81
*SLP1*	0.80	0.78	1.01	0.64	0.62
*CITED4*	0.44	0.62	0.52	0.39	0.24
*EGFR*	0.22	0.53	0.30	0.30	0.18
*CDK8*	0.73	1.13	0.62	0.42	0.23
*HNRPDP*	0.67	0.74	0.20	0.10	0.10
*GMPS1*	0.86	0.60	0.92	0.41	0.35
*GSTA3*	0.77	0.44	0.79	0.52	0.53
*MAP4*	0.49	0.59	0.54	0.73	0.41
*MAP2*	0.28	0.57	0.44	0.46	0.57
*HNRPDP*	0.67	1.04	0.20	0.10	0.10
*GSTA3*	0.77	0.44	0.79	0.52	0.53
*FosL2*	0.37	0.46	0.50	0.13	0.24
*BLK*	0.73	0.98	0.37	0.87	0.65
*RRAS2*	0.63	0.95	0.62	0.57	0.32
*PAK6*	0.78	0.91	0.69	0.42	0.30
*CASP7*	0.66	0.81	0.54	0.56	0.23
*ELF2*	0.54	0.61	0.36	0.19	0.16
*BMP4*	0.53	0.84	0.42	0.24	0.14
*DYRK2*	0.36	0.50	0.05	0.10	0.10
*MAX*	0.64	0.72	0.49	0.27	0.24
*PTPN13*	0.48	0.73	0.42	0.47	0.22
*ETS2*	0.67	0.70	0.50	0.49	1.09

In order to experimentally test whether significant gene binding by Egr1 was associated with expression changes that were Egr1-dependent *in vivo*, small interfering RNA (siRNA) to Egr1 (SiEgr1) was used to 'knock down' Egr1 expression in M12 cells (Figure 3c, lane 4). Transcript levels of 14 representative genes and *Egr1 *were measured by qRT-PCR in UV-stimulated M12 cells with or without prior silencing of Egr1. Two genes (*PSD3 *and *Egr2*) that exhibited positive expression changes and seven genes (*EGFR*, *RRAS2*, *PAK6*, *ELF2*, *DYRK2*, *MAX *and *CASP7*) that exhibited decreased mRNA expression upon UV stimulation were reversed in expression upon Egr1 silencing (Figure 3d), and one gene, *BLK*, was further repressed upon Egr1 silencing. Four genes (*IL11RA*, *H3A*, *IGFBP6 *and *SCAP2*) showed no change. Thus, the expression of at least 10/14 target genes (Figure 3d) was Egr1-dependent. These observations provide strong experimental support for the conclusion that UV-induced Egr1 promoter binding is associated with regulation of transcription. In summary, of the 25 genes that were validated by conventional ChIP, 18 were also validated as functional by the effects on gene expression using qRT-PCR analysis (hence, 18/37 genes that were shown to have significant transcript level changes were also shown to have Egr1 bound to their promoters). The 14 genes on which the siRNA experiment was performed were all from the 37 genes that were validated by qRT-PCR analysis and this set was chosen as its members exhibited increased expression and define excellent targets for siRNA testing. The siRNA results support the conclusion that Egr1 is specifically bound to and regulates expression of these genes.

### UV-C stimulation increases phosphorylation of EGFR and inhibitors of EGFR block Egr1 expression

**Figure 4:**
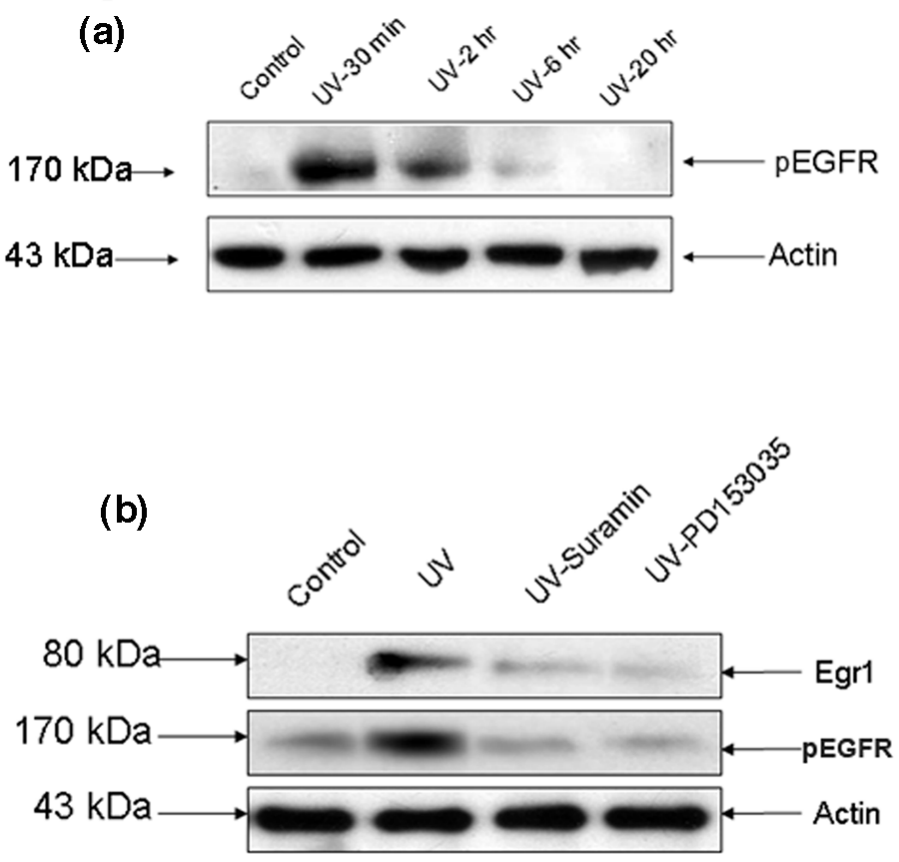
EGFR activation upon UV stimulation. **(a) **After 40 J/m^2 ^UV-C radiation, 100 μg of the protein lysate was used for immunoprecipitation with EGFR antibody and this sample was used for immublotting with pTyr antibody showing activated EGFR. pEGFR, phosphorylated EGFR. **(b) **Western blot analysis of M12 cells treated with suramin or PD153035 for 45 minutes followed by UV-C irradiation.

We have previously shown in other cells that UV irradiation leads to rapid activation of EGFR, activation of the ERK pathway, and to a large induction of Egr1 expression [21]. Similarly, in M12 cells we observed that ERK1/2 inhibitors block UV induction of Egr1 (Figure 1c). Phosphorylated EGFR was greatly increased 30-120 minutes after UV irradiation, as demonstrated by immunoprecipitation using EGFR antibody followed by western analysis using an anti-p-tyrosine antibody (Figure 4a).

Egr1 expression observed here is downstream of the activated phosphorylated EGFR in UV-stimulated M12 cells, as shown by the treatment of cells with PD153035 prior to UV-C irradiation. In addition, since UV irradiation commonly stimulates autocrine activation of EGFR by liberation of heparin-binding growth factors [27], we also pretreated the cells with suramin. Treatment with PD153035 inhibited Egr1 expression by approximately 85% and suramin inhibited Egr1 expression by approximately 80% (2 h; Figure 4b). In addition, our 'ChIP-on-chip' results showed that EGFR expression was suppressed by Egr1 upon UV irradiation (Table 1) and increased by threefold when the cells were irradiated after silencing Egr1 expression. The result indicates that Egr1 promoter binding is specifically associated with decreased transcription of EGFR, suggesting the presence of a negative feedback loop controlling EGFR expression by Egr1.

### Egr1 over-expression after UV irradiation leads to growth inhibition and apoptosis

**Figure 5:**
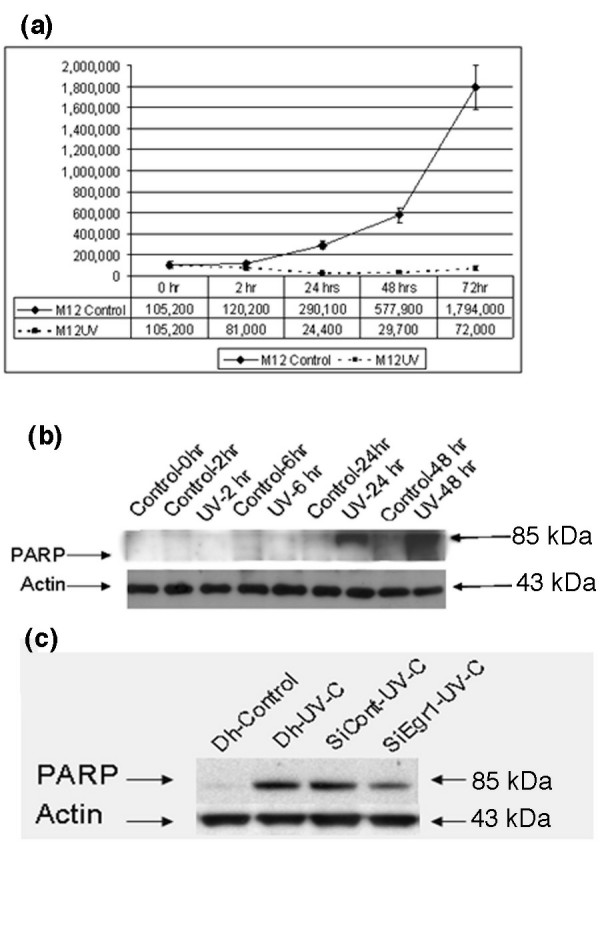
Role of Egr1 in apoptosis. **(a) **Growth curve of M12 cells over a period of 72 h, with or without UV-C irradiation. The experiments were performed in triplicates and the error bars represent the data from the triplicate experiments **(b) **Western blot analysis of M12 cells treated with UV-C, and collected at varying time points after UV irradiation. Anti-PARP (to demonstrate apoptosis) was used to identify PARP cleavage product (this antibody only detects the cleaved product and not the native protein). **(c) **M12 cells were treated with SiGenome control and SiEgr1 and followed by UV-C stimulation. Cells were collected after 24 h of UV treatment, and western blot analysis, using anti-PARP, showed that Egr1 is involved in apoptosis of M12 cells.

UV stimulation promotes apoptosis in a variety of cell types. We therefore examined the growth and survival properties of M12 cells following UV stimulation by direct proliferation measurements over 3 days. Untreated M12 cells in standard medium grew rapidly to high density whereas cells treated by UV irradiation were drastically retarded in growth, which was apparent within 24 h (Figure 5a). By 24 h numerous detached and floating cells and extracellular debris were apparent, suggesting apoptosis in these cells. A Poly(ADP)-ribose polymerase (PARP) assay revealed a high proportion of PARP degradation, indicating apoptosis, whereas no degradation was apparent in untreated cells (Figure 5b). Cell numbers were reduced 25-fold compared to control cells at 72 h after treatment. These results indicate that EGFR activation leads to apoptosis in M12 prostate cells.

To test whether apoptosis of M12 cells was Egr1 dependent *in vivo*, M12 cells were treated with siEgr1 to silence Egr1 expression for 48 h followed by UV-C. Egr1 mRNA and protein expression was effectively silenced by this treatment (Figure 3c). Cells were collected 24 h later and the PARP assay demonstrated that cells underwent reduced apoptosis in the absence of Egr1, clearly showing that Egr1 is an important mediator of UV-C-induced apoptosis (Figure 5c). These results confirm the role of Egr1 as a mediator of the apoptosis response.

## Discussion

### Egr1 binds a large spectrum of promoters that result in transcriptional regulation

We examined the role of Egr1 in UV-irradiated tumorigenic human M12 prostate cancer cells. Our data show that Egr1 binds to a surprisingly large number of promoters (283 promoters) of an array containing approximately 10,012 unique proximal promoter sequences. Several of our observations suggest that Egr1 promoter binding contributes to the regulation of gene expression in UV-treated cells. First, 5.2% (15/288) of the significantly bound genes are known to interact with Egr1 and most of them (11 genes) are known to be regulated by Egr1 (Figure S3 in Additional data file 4). For example, *DMRT1 *and *EGFR *are both shown to be direct targets of Egr1 and Egr1 binds to their promoters. Second, a majority of the 283 promoter sequences contain consensus EBSs (58.3%). Twenty-five genes were examined by conventional ChIP and the results support the conclusion that ChIP-on-chip can be used to identify targets and with low false discovery rates. Gene expression studies by qRT-PCR and Affymetrix expression analysis show that promoter binding leads to significant gene expression changes of the target genes. The qRT-PCR experiments were also done in the very widely used DU145 prostate cancer cell line, which also over-expresses Egr1 upon UV irradiation (Table S3 in Additional data file 3). The results comparing the two cell lines clearly show that the gene expression pattern of most of the target genes remained the same across the two cell lines, thus showing that most of the gene expression changes in the target genes were identical. Prior treatment with siRNA to silence Egr1 expression *in vivo *reversed the expression of Egr1 target genes, clearly supporting the role of Egr1 as a functional transcription factor in M12 prostate cancer cells. These results are consistent with the conclusion that promoter arrays have accurately revealed the identity of 288 genes that are significantly bound by Egr1 upon UV irradiation. The results further suggest that at least 40% of the bound promoters involve DNA binding sequences that have not been recognized previously.

### Egr1 expression is downstream of the EGFR signaling pathway and negatively regulates EGFR

We and others have shown that a major mechanism leading to the expression of Egr1 is via activation of EGFR and the ERK1/2 pathway [28]. We show that the same mechanism applies to human prostate M12 cells following UV irradiation, where Egr1 expression was blocked by inhibitors of EGFR, ERK1/2 and suramin. This indicates that heparin-binding EGF-like ligands may be released from the irradiated cells and participate in the activation of EGFR, consistent with previous observations from normal mouse cells [21] and immortalized human keratinocytes [29]. Our study also demonstrates that EGFR itself is a target of Egr1, which leads to suppression of its transcription and decreased protein expression. We show that EGFR activated by UV stimulation induces Egr1, which serves to limit the production of EGFR and thereby blocks its continued activation and signaling. Interestingly, the *MAX *gene was also identified as a target of Egr1 and its expression was repressed in UV-irradiated cells. Perini *et al. *[30] showed that the MAX protein dimerizes with n-myc and this heterodimer binds to the *EGFR *promoter and affects its transcription. Our results clearly demonstrate that after UV irradiation, Egr1 is significantly bound to the promoters of both *EGFR *and *MAX *and the gene expression for both is suppressed, thus supporting the concerted action of the two genes [30]. Another indication of this concerted action comes from the observation that MMP9 mediates EGFR transactivation by G-protein-coupled receptors and, in our dataset, MMP9 is also down-regulated [31]. Therefore, ChIP-on-chip clearly identifies several genes that are reported to work concordantly to serve a similar function. Also in the present study, our results show that Egr1 is a transcriptional repressor for a number of its target genes. Egr1 has predominantly been discussed as a transcriptional activator by most groups, including ours, but this is the first comprehensive study of the identification of Egr1 target genes on a high throughput scale. These results clearly indicate that Egr1 can act as both a transcriptional activator as well as a repressor protein.

### Egr1 mediates UV- induced apoptosis

The most notable physiological change observed in response to UV irradiation of M12 cells is apoptosis. Egr1 promotes apoptosis in UV-C-irradiated mouse NIH3T3 cells or mouse HC11 epithelial cells [21,32]. Similar to previous findings, we observed apoptosis in M12 prostate cancer cells in response to UV irradiation. Here we observed that Egr1 over-expression mediates UV-induced apoptosis and this response is blocked by silencing Egr1 expression using siRNA. Several of the Egr1 target genes identified by ChIP-on-chip have a previously demonstrated role in apoptosis. These include *TNFSF6*/*CD95L *(*FasL*), *FAP1 *and *fosL2*. FasL is pro-apoptotic and is significantly up-regulated after UV irradiation in our cells and FAP1/PTPN13, which prevents apoptosis, is significantly down-regulated in our cell system, thus showing that the Egr1 function in apoptosis occurs through its downstream targets. Other apoptosis related genes that were bound by Egr1 include *Bcl G*, *BLK*, *BMF*, *CASP7*, *TNFRSF19L*, and *TNFSF5*. Most are mediators of the classic apoptosis pathway. Moreover, it has been shown previously that TNFSF6/CD95L induces reactive oxygen intermediate formation that, in turn, activates the src family kinase 'Yes', which rapidly associates with and phosphorylates EGFR. Activated EGFR triggers CD95-tyrosine phosphorylation, which is a signal for membrane targeting of the EGFR/CD95 complex, and subsequently recruits the Fas-associated death domain and induces apoptosis [33]. Further, CD95L-induced cell death is enhanced by EGFR inhibition [34], which is exactly what we see in our cells, and both the genes encoding these proteins are identified as Egr1 targets by the present study. Conversely, inhibition of expression and/or the transcriptional activity of Egr1 and Egr3 are known to repress FasL activation [35], suggesting that Egr1 is essential for FasL expression. These observations indicate that UV-induced Egr1 expression may lead to apoptosis through stimulation of the classic TNF/CD95-initiated pathway of apoptosis and not through the p53/p73 pathway (Figure S4 in Additional data file 4). Previous reports from our laboratory have shown that, under normal growth conditions, Egr1 is required for growth and proliferation of prostate cancer cells [13,36]. Conversely, in the present study we observe that when prostate cancer cells are UV irradiated, Egr1 functions in inducing apoptosis of these cells. Our group and others have shown earlier that Egr1 can undergo several post-translational modifications, such as phoshorylation (approximately 25% of Egr1 is made up of Ser/Thr), acetylation and sumoylation [37]. It has also been shown previously that the active form of Egr1 protein produced by UV induction is highly phosphorylated, in contrast to the Egr1 induced by serum, growth factors, or 12-O-tetradecanoylphorbol-13-acetate (TPA). The nature of the phosphorylated forms of Egr1 has not yet been analyzed, but phosphorylated forms bind to DNA more efficiently [37,38]. Therefore, we hypothesize that the differential post-translational modifications of this protein enable it to function in several different pathways depending on the stimulus that induces its expression. Also, our group has previously shown that p53 is a target of Egr1 and is responsible, in turn, for the role of Egr1 as a pro-apoptotic protein [38]. For our present study we used M12 prostate cancer cells, which are SV40 T antigen transformed and, hence, there is very little unbound native p53 available in them. Therefore, it was not surprising that the gene expression of p53 after UV induction did not show much change. In addition, we also did not see changes in gene expression for p73 and PTEN transcripts. Therefore, it seems that the p53/p73/PTEN pathways are not very active in these cells, consistent with the epigenetic suppression commonly observed for these genes in prostate cancer, whereas Egr1 does induce the expression of pro-apoptotic genes, such as *TNFSF6*, which are responsible for its apoptotic response in these cells. Previous studies have shown that the pro-apoptotic protein 'Bax' undergoes polymerization and then translocates to the mitochondrial membrane, leading to mitochondrial membrane depolarization and liberation of nuclease activity but not cytochrome c [39]. Here, we identified that the Bax receptor, TOM22, is a target of Egr1, which is over-expressed in our UV-treated cells. This protein is a translocase of the outer membrane of mitochondria and acts as a receptor for BAX Halpha1, which is an important pro-apoptotic protein [40] that may act to facilitate a Bax-dependent apoptosis analogous to the mechanism observed in UV-stimulated keratinocytes. Hence, by over-expression of TOM22, 'Bax' signaling leads to enhanced apoptosis. Another target gene, *TC21 *(*RRAS2*), is known to mediate transformation and cell survival via the activation of the Phosphoinositide 3-kinase (PI3K)/AKT and Nuclear factor κB (NFκB) signaling pathway [41], and this gene is down-regulated in our data set, which is in accordance with the role of Egr1 in growth inhibition. Affymetrix gene expression analysis also identified several other apoptosis related genes in the list of significantly deregulated genes, such as c-*jun*, *junD*, *fosB*, *TNFSF9*, and *TNFSF13*, which might play important roles in the apoptosis pathway after UV irradiation. In addition, EGFR, which has a proven role in proliferation of cells, was also inhibited by Egr1, reinforcing the role of Egr1 in growth inhibition. Our data clearly show that Egr1 is a mediator of transcription of numerous pro-apoptotic genes, which may work concordantly in UV-stimulated prostate cancer cells. Therefore, all these target genes concordantly function in leading to Egr1-dependent apoptosis and growth inhibition. In conclusion, this is the first comprehensive study to identify approximately 283 targets of Egr1 with the aid of high throughput ChIP-on-chip analysis. We have shown that, upon UV stimulation, prostate cancer cells undergo Egr1-dependent apoptosis and this function of Egr1 is mediated by at least several of the newly identified target genes.

## Conclusion

**Figure 6:**
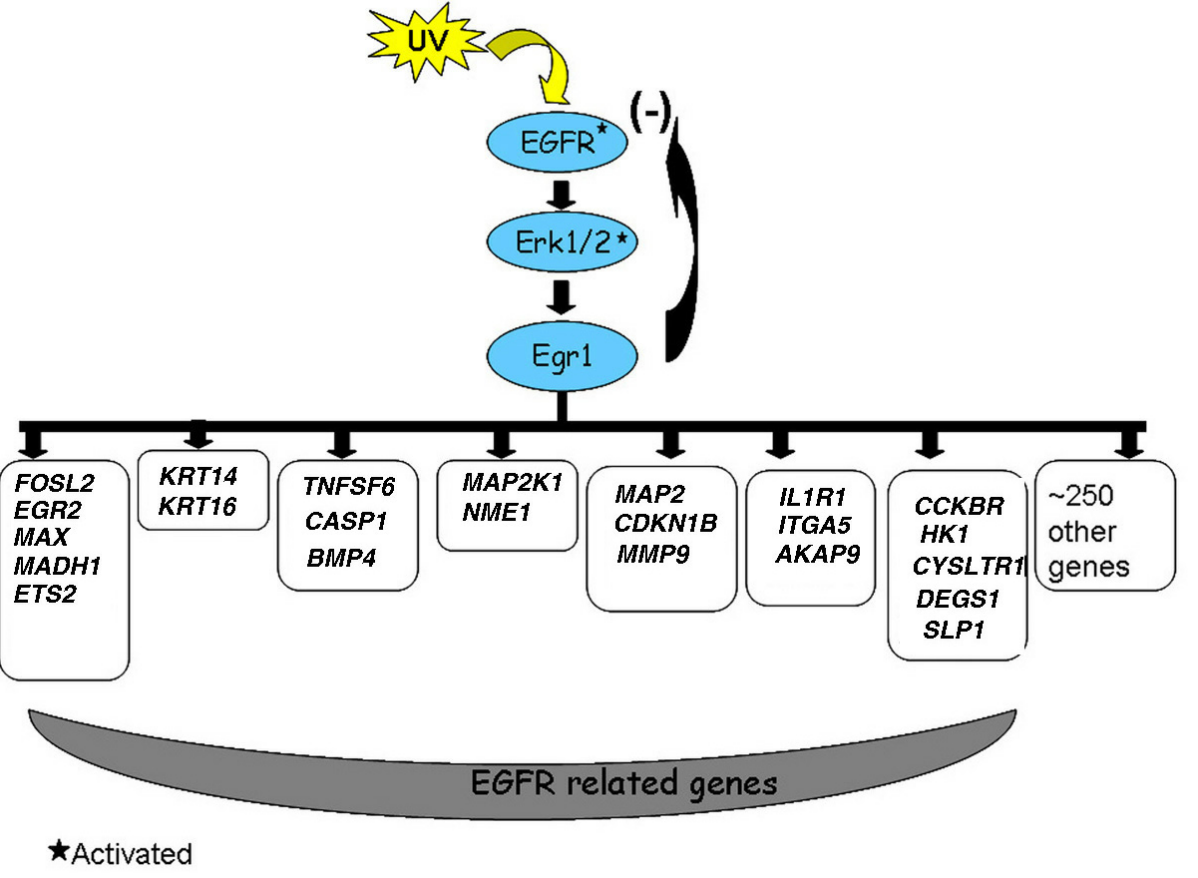
Schematic diagram of the activation of Egr1 and the identification of its downstream targets upon UV simulation.

We have shown that UV irradiation of prostate cancer cells leads to rapid and extensive induction of Egr1 via activation of the EGFR/ERK1/2 pathway and to apoptosis. Using ChIP-on-chip, we observed that the increased Egr1 binds to an extensive profile of over approximately 288 promoters. We confirmed that promoter binding corresponds to the regulation of gene expression for many of these target promoters. The expression of at least 23 of the target genes are known to be correlated with activation of EGFR. Moreover, this report demonstrates that EGFR itself is a target of Egr1 and Egr1 inhibits its expression, suggesting a negative feedback loop in order to limit EGFR expression and, hence, its function (Figure 6). Egr1 also binds to a panel of apoptotic factors, leading to alteration of their transcript levels, and siRNA experiments confirm that Egr1 is essential for the induction of these factors and for apoptosis. We propose that the newly identified targets might play a role in the EGFR-promoted apoptotic response and provide an explanation of the role of Egr1 in UV-irradiated cells.

## Materials and methods

### Cell culture and treatment

Prostate P69 cells are low-tumorigenic, SV40-Tag transformed human epithelial, prostate cells and M12 cells are a metastatic cancerous derivative of P69 [23]. The M12 cells used here were a gift of S Plymate and are insensitive to androgen. They were cultured as described previously [37]. For UV-C irradiation (40 J/m^2^) the medium was removed and collected in separate tubes, cells were then irradiated in a UV-Stratalinker (Stratagene, la Jolla, CA, USA) and the collected medium was then added back to them. For mock treatment, the growth medium was collected in separate tubes and then added back after the UV treatment was completed in the parallel set. Apoptosis in cells treated with UV-C was detected using anti-PARP antibody from Sigma (P1991-1VL, St. Louis, MO, USA). Suramin and EGFR inhibitor (PD153035) were obtained from Calbiochem (San Diego, CA, USA). ERK1/2 inhibitor (U0126) was obtained from Promega (Madison, WI, USA).

### Western blot analysis

Western blot analyses were performed as described [24]. Antibodies against Egr1 (C-19), Egr1 (588), p-Tyr (PY-99; sc-7020) and EGFR (1005; sc-03) were rabbit polyclonals from Santa Cruz Biotechnology (Santa Cruz, CA, USA). Phospho p44/42 MAPK monoclonal antibody was obtained from Cell Signaling Technology, Inc. (Beverly, MA, USA). Anti-β-actin antibody was a mouse monoclonal antibody from Sigma. The images were quantified using image J software from NIH.

### Cell proliferation assay

A day prior to the experiment, cells were seeded in triplicate into 6- well plates. At day 0, cells were treated with UV-C and later harvested for counting, and protein and total mRNA extraction. This procedure was repeated each day after treatment according to a time course from day 0 to day 6. Cells were counted using a Beckman Coulter Counter, Z2 (Fullerton, CA, USA). Cell proliferation was also assessed by plating approximately 1,000 cells in each well of a 96-well plate followed by UV-C treatment the next day. From day 2, plates were analyzed daily using WST1 assay according to the manufacturer's instructions (Roche Diagnostics, Mannheim, Germany). Relative cell numbers were calculated as the change in proliferation compared to control wells at each time point.

### Chromatin immunoprecipitation

M12 prostate cancer cells were used for ChIP as previously described [24,42]. Briefly, 2 × 10^7 ^cells were fixed with formaldehyde, neutralized with glycine and rinsed with cold phosphate-buffered saline. After lysis, samples were sonicated to an average DNA length of 1,000 bp (Figure 2). Immunoprecipitation of 2 mg pre-cleared chromatin was carried out by addition of 6 μg of anti-Egr1 (sc-110) antibody and anti-rabbit IgG antibody (sc-2027, used as a negative control). Two independent ChIP experiments were performed for each antibody. The purified ChIP-captured DNA of samples and the total input DNA consisting of genomic DNA prepared from control cross-linked cells were amplified using the Round A/B/C random amplification of DNA protocol [43].

### Promoter array hybridization, data analysis, statistics and criteria of significance

The promoter arrays with about 12,000 human promoters spotted in triplicate have been described in our previous papers as well as in the supplemental Materials and methods (Additional data file 1) [44,45]. Hybridization and data analysis were essentially carried out as described in our previous papers and as described in the supplemental Materials and methods (Additional data file 1) [24,43]. Significant differential hybridization between UV and mock-treated control samples were defined as fold change >1.4 and with *p *< 0.005. Functional relationships and potential regulatory relationships among gene products were identified using Pathway studio 5.0 of Ariadne Inc. (Rockville, MD, USA) and Ingenuity Pathway Analysis of Ingenuity Systems Inc. (Redwood City, CA, USA). Statistical analyses were essentially as described in our previous paper [24] and were performed using the Limma package in BioConductor and the R program [46]. M-A plots were constructed where:

M = log_2 _R - log_2 _G

and

A = (log_2 _R + log_2 _G)/2

where R is the intensity of the scanner output signal for the experimental sample fluorophore (channel 2, Ch2), and G is the scanner output signal for the reference sample fluorophore (channel 1, Ch1) on the background-subtracted, normalized, and scaled channel intensities. B statistics [46], and Chi-squared test with Yates criteria were calculated as implemented in the R program. B is equivalent to a 'penalized' t-statistic:

T = <M>/√[(a + s^2^)/n]

where 'a' is the penalty estimated from the mean of M-values (<M>), and standard deviation of the sample variances (s^2^). Random genes were chosen from the promoter array for comparison with our significantly detected gene list. For this, we used command 'sample' in the R program to randomly select 200 or 1,000 numbers from 1 to 12,000 without replacement, where 12,000 is the total number of genes represented on the array and the corresponding genes are the 1,000 random genes. Chi square and Fisher exact test were done using the R program.

### Microarray expression analysis

All microarray expression analyses were performed in duplicate using GeneChip^® ^U133 Plus 2 arrays (Affymetrix, Inc., Santa Clara, CA, USA) as described [24]. Statistical analysis was carried out with the aid of the Cyber-t software [47]. The analysis module computes regularized *t*-tests using a Bayesian estimate of the variance among the gene measurements to infer significant gene changes; *p *< 0.001 genes were accepted as differentially expressed.

### Validation of gene expression by qRT-PCR

qRT-PCR using Sybr Green (Applied Biosystems, Carlsbad, CA, USA) was performed as described previously to verify ChIP-Chip microarray analysis as well as to measure the gene expression changes of the target genes [24,43,46]. To validate the promoter array results, primers for 25 genes were designed such that the amplicons have at least one putative Egr1 binding site identified by the TF SEARCH program TESS [48]. PCR primers of the genomic regions were designed using the IDT Primer quest software (IDT technologies, Coralville, IA, USA) (primer sequences can be provided on request). For gene expression studies, primers were designed in the exon regions of the genes and the *GAPDH *gene was used as an internal control. The relative quantification was given by the Ct values, determined for triplicate reactions for test and reference samples for each target and for the internal control gene (*GAPDH*). Relative expression level was determined as 2^-ΔΔCt^, where ΔΔCt = ΔCt (target sample) - ΔCt (reference sample).

### siRNA and transfection

siRNA against Egr1 was obtained from Dharmacon (Lafayette, CO, USA). Briefly, four pooled siRNA duplexes were transiently transfected into M12 prostate cancer cells following the Dharmacon protocol using Dharmafect reagent 1. Mock transfection was done in parallel using SiGenome control (Dharmacon) as negative control. The concentration of siRNA used was standardized to get maximum knockdown without affecting the viability of the cells. To study the effect of siRNA on downstream targets of Egr1, cells were treated with UV (40 J/m^2^) 48 h after the transfection, and RNA isolation was done 2 h after UV treatment as described.

## Abbreviations

ChIP: chromatin immunoprecipitation; EBR: Egr1 binding site; EGFR: epidermal growth factor receptor; Egr1: Early growth response-1; PARP: Poly(ADP)-ribose polymerase; qRT-PCR: quantitative real time PCR; siRNA: small interfering RNA; UV: ultraviolet.

## Authors' contributions

SA was involved in the design of the study; she carried out the experiments and wrote the manuscript. YW and ZJ carried out the statistical analyses. SVS helped with the experiments and writing the manuscript. AM did the experiments, KSD helped with the initial bioinformatic analysis, MB helped in the designing and construction of the promoter arrays, and MMCC helped with the designing of the promoter arrays and writing the manuscript. EA and DM conceived the study and participated in its design, coordination and writing the manuscript. All authors read and approved the final manuscript.

## Additional data files

The following additional data are available with the online version of this paper. Additional data file 1 describes of the construction of the promoter arrays, hybridization and analysis of the promoter arrays. Additional data file 2 includes Table S1, which lists information on each of the identified targets of Egr1. Additional data file 3 includes Tables S2 and S3. Additional data file 4 contains supplemental Figures S1-S4.

## Supplementary Material

Additional data file 1 Construction of the promoter arrays, hybridization and analysis of the promoter arrays. Click here for file

Additional data file 2 Identified targets of Egr1. Click here for file

Additional data file 3 Table S2: log fold changes and *p*-values of the Egr1 target genes that were significantly differentially expressed between UV treated and mock cells using Affymetrix HGU133plus 2 arrays. Table S3: the gene expression analysis of Egr1 target genes using qRT-PCR of RNA extracted at various time points after UV-C treatment of DU145 cells. Click here for file

Additional data file 4 Figure S1: diagram showing previously reported interactions between EGFR and 23 other Egr1 target genes using Pathway Studio (Ariadne Inc.). Figure S2: plot showing the distribution of fold change values from Affymetrix gene expression data and M values from ChIP-on-chip data for Egr1 target genes. Figure S3: diagram showing previously reported interactions between Egr1's target genes using Pathway Studio (Ariadne Inc.). Figure S4: gene expression of *p53*, *p73*, *TGFb1 *and *PTEN *was studied by qRT-PCR analysis of RNA isolated from M12 cells at various time points after UV-C treatment. Click here for file
