# Supplementary material for: Egr1 regulates the coordinated expression of numerous EGF receptor target genes as identified by ChIP-on-chip
Source: Genome Biol. 2008 Nov 25;9(11):R166. doi: 10.1186/gb-2008-9-11-r166 (PMC2614498; doi:10.1186/gb-2008-9-11-r166)
Supplement: Additional data file 1 — Construction of the promoter arrays, hybridization and analysis of the promoter arrays. [file gb-2008-9-11-r166-S1.doc]

**Supplemental Materials and Methods**

**Design and Fabrication of Human Promoter Microarray**

Human promoter sequences (1000bp upstream and 500bp downstream from transcription initiation site) were utilized for fabrication of the arrays. Approximately 5000 sequences were retrieved batch-wise from GoldenPath. Primers for amplification of these sequences were generated batchwise using Primer 3 designed to generate an average amplicon of 1200 nts. An additional ~ 13,000 sequences were prepared using the primer set developed by the Whitehead Institute Center for Microarray Technology designed to generate average amplicons of ~840 nts. The promoter sequences were amplified, purified and spotted onto UltraGAPS coated slides (Corning Incorporated, Corning, NY) in the presence of 50% DMSO. The products of all amplifications were visualized by agarose gel electrophoresis. Over 70% of all amplifications yielded single products of the expected size. Failed amplifications were recorded and ignored in the subsequent robot-assisted selection for spotting using Biomek FX (Beckman Coulter, Inc). Promoter arrays were printed on two slides, set A and set B. Each array contains triplicates of 6400 spots. In total, the promoter microarray contains 12186 promoter sequences (including 192 oligos), 544 non-promoter controls, 32 salmonella controls, 32 Cy Dye controls and 6 blank spots. Many of the promoters on the array are from genes of particular relevance to cancer and the array includes promoters from most of the genes that are known to be regulated by methylation in cancer. These human promoter arrays have been used for high-throughput DNA methylation assay and ChIP-on-Chip analysis.

**Promoter Array Hybridization and Data Analysis**

The promoter arrays with about 12,000 human promoters spotted in triplicates and have been described in our previous papers as well as in the supplemental materials and methods. Hybridization and data analysis was carried out as follows. ChIP-captured and PCR-amplified products were purified by the MinElute PCR purification kit (QIAGEN), and 2 μg of ChIP UV sample and 2 μg of Control sample were labeled in parallel with the fluorophore Cy3-dCTP or Cy5-dCTP using Ready-To-Go DNA Labeling Beads (Amersham Biosciences). To assess the reproducibility of the experiment, two independent cell samples were processed through the ChIP, amplification, and conjugation procedure in parallel. Two additional arrays were used for each sample but with the order of conjugation of dyes reversed (“dye switching”). Thus, 4 × 3 replicate hybridizations were carried out per sample. Hybridization was preformed 18 h at 42°C. Slides were washed and scanned with a Perkin Elmer Scanarray Express Microarray scanner (PerkinElmer Inc.). Microarray data were retrieved with Quantarray Microarray Analysis software (PerkinElmer Inc.). Data reduction and preliminary analysis were carried out using the Limma package with print-tip lowess normalization, and followed by between-array scale normalization. In order to establish statistical thresholds, medians were calculated from the triplicate values of each slide, and the medians of the four hybridizations per sample for UV -treated cells were compared to the four values from the mock-treated control cells.

**Supplemental Results**

**Egr1 does not regulate Egr1-suppressor gene target promoters in M12 cells.**

Egr1 is a direct and positive regulator of key tumor suppressor genes such p53, p73, and PTEN in certain cells. We therefore examined the RNA expression of these gene products following UV irradiation of M12 by quantitative PCR. No significant increases in transcript levels were observed for any of these gene transcripts. We examined the expression of transcripts at various time points (***cf.* Figure 1**) following stimulation, and no deviation from basal levels was observed at any time point examined. In contrast, the growth factor TGFβ1, which has been reported to be a positive growth factor for M12 cells, exhibited a consistent elevation which was maximal at 6 hours (**Supp. Figure S3)**. These results provide evidence that Egr1 does not regulate the tumor suppressor genes p53, p73 and PTEN in tumorigenic M12 prostate cells, but are consistent with previous observations that Egr1 is a positive regulator the TGFβ1 gene. Moreover, the results suggest that regulation of the p53, p73, and PTEN genes by Egr1 is not part of the apoptosis response observed here.
