# Supplementary material for: Egr1 regulates the coordinated expression of numerous EGF receptor target genes as identified by ChIP-on-chip
Source: Genome Biol. 2008 Nov 25;9(11):R166. doi: 10.1186/gb-2008-9-11-r166 (PMC2614498; doi:10.1186/gb-2008-9-11-r166)
Supplement: Additional data file 2 — Identified targets of Egr1. [file gb-2008-9-11-r166-S2.doc]

**Supplemental Table S1:** The table lists all the genes whose promoters were significantly detected. The various columns show the details of these genes after the review of literature.

|  | **Symbol** | **Description** | Known UV response | Known EGFR interaction | Known involvement in P Ca | Conventional IP validation | q-RTPCR validation |
| --- | --- | --- | --- | --- | --- | --- | --- |
| NM_000269 | NME1 | nucleoside-diphosphate kinase 1 isoform a | yes | yes | yes | yes | yes |
| NM_002382 | MAX | MAX protein | yes | yes | no | yes | yes |
| NM_001202 | BMP4 | bone morphogenetic protein 4 preproprotein | yes | yes | yes | yes | yes |
| NM_005253 | FOSL2 | FOS-like antigen 2 | yes | yes | no | yes |  |
| NM_005228 | EGFR | epidermal growth factor receptor isoform d | yes | yes | yes | yes | yes |
| NM_000188 | HK1 | hexokinase 1 isoform HKI | yes | yes | no |  |  |
| NM_004994 | MMP9 | matrix metalloproteinase 9 | yes | yes | yes |  |  |
| NM_033294 | CASP1 | caspase 1 | yes | yes | no |  |  |
| NM_004064 | CDKN1B | cyclin-dependent kinase inhibitor 1B | yes | yes | no |  |  |
| NM_005239 | ETS2 | Homo sapiens v-ets erythroblastosis virus E26 oncogene homolog 2 | yes | yes | no |  |  |
| NM_002755 | MAP2K1 | mitogen-activated protein kinase kinase 1 | yes | yes | no |  |  |
| NM_005597 | NFIC | nuclear factor I/C (CCAAT-binding transcription factor) | yes | yes | no |  |  |
| NM_000639 | TNFSF6 | apoptosis (APO-1) antigen ligand 1 | yes | yes | no |  |  |
| NM_005751 | AKAP9 | A-kinase anchor protein 9 isoform 4 | no | yes | no |  |  |
| NM_006639 | CYSLTR1 | cysteinyl leukotriene receptor 1 | no | yes | no |  |  |
| NM_144780 | DEGS1 | degenerative spermatocyte homolog 1, lipid | no | yes | no |  |  |
| NM_000526 | KRT14 | keratin 14 (epidermolysis bullosa simplex, Dowling-Meara, Koebner) | no | yes | yes |  |  |
| NM_005900 | MADH1 | MAD, mothers against decapentaplegic homolog 1 (Drosophila) | no | yes | no |  |  |
| NM_000731 | CCKBR | cholecystokinin B receptor | no | yes | no |  |  |
| NM_000877 | IL1R1 | interleukin 1 receptor, type I | no | yes | no |  |  |
| NM_002205 | ITGA5 | integrin, alpha 5 (fibronectin receptor, alpha polypeptide) | no | yes | no |  |  |
| NM_003064 | SLPI | secretory leukocyte protease inhibitor (antileukoproteinase) | no | yes | no |  |  |
| NM_002374 | MAP2 | microtubule-associated protein 2 isoform 4 | no | yes | no |  |  |
| NM_005557 | KRT16 | keratin 16 | no | yes | no |  |  |
| NM_001260 | CDK8 | cyclin-dependent kinase 8 | yes | no | yes | yes | yes |
| NM_012250 | RRAS2 | related RAS viral (r-ras) oncogene homolog 2 | yes | no | no | yes | yes |
| NM_080683 | PTPN13 | protein tyrosine phosphatase, non-receptor type | yes | no | no | yes |  |
| NM_030885 | MAP4 | microtubule-associated protein 4 isoform 3 | yes | no | yes | yes | yes |
| NM_002715 | SPK1A | S-phase kinase-associated protein 1A isoform b | yes | no | no | yes |  |
| NM_014417 | BBC3 | Bcl-2 binding component 3 | yes | no | yes | yes | yes |
| NM_033503 | BMF | Bcl2 modifying factor isoform bmf-3 | yes | no | no | yes |  |
| NM_002512 | NME2 | non-metastatic cells 2, protein (NM23B) expressed in | yes | no |  | yes | yes |
| NM_001227 | CASP7 | caspase 7, apoptosis-related cysteine protease | yes | no | yes | yes | yes |
| NM_002107 | H3F3A | H3 histone, family 3A | no | no | no | yes | yes |
| NM_006569 | CGR11 | cell growth regulatory with EF-hand domain | no | no | no | yes |  |
| NM_006874 | ELF2 | E74-like factor 2 (ets domain transcription factor) | no | no | no | yes | yes |
| NM_000399 | EGR2 | early growth response 2 (Krox-20 homolog, Drosophila) | no | no | no | yes | yes |
| NM_138724 | BCLG | apoptosis regulator BCL-G isoform 3 | no | no | yes | yes |  |
| NM_020168 | PAK6 | p21-activated protein kinase 6 | no | no | yes | yes | yes |
| NM_030667 | PTPRO | receptor-type protein tyrosine phosphatase O | no | no | no | yes | yes |
| NM_133467 | CITED4 | Cbp/p300-interacting transactivator, 4 | no | no | no | yes | yes |
| NM_001715 | BLK | B lymphoid tyrosine kinase | no | no | no | yes | yes |
| NM_003875 | GMPS | guanine monophosphate synthetase | no | no | no | yes | yes |
| NM_006435 | IFITM2 | interferon induced transmembrane protein 2 (1-8D) | yes | no | no |  | yes |
| NM_004512 | IL11RA | interleukin 11 receptor, alpha | no | no | yes |  | yes |
| NM_003583 | DYRK2 | dual-specificity tyrosine-(Y)-phosphorylation regulated kinase 2 | no | no | no |  | yes |
| NM_000847 | GSTA3 | glutathione S-transferase A3 | no | no | no |  | yes |
| NG_000874 | HNRPDP | heterogeneous nuclear ribonucleoprotein D | no | no | no |  | yes |
| NM_020243 | Tom22 | mitochondrial import receptor Tom22 | no | no | no |  | yes |
| NM_015310 | PSD3 | ADP-ribosylation factor guanine nucleotide factor 6. | yes | no | no |  | yes |
| NM_006569 | CGR11 | cell growth regulatory with EF-hand domain | no | no | no |  |  |
| NG_001025 | PPIP9 | peptidylprolyl isomerase (cyclophilin) pseudogene 9 | yes | no | no |  |  |
| NM_006048 | UBE4B | ubiquitination factor E4B (UFD2 homolog, yeast) | yes | no | no |  |  |
| NM_015840 | ADAR | adenosine deaminase, RNA-specific isoform ADAR-b | yes | no | no |  |  |
| NM_001123 | ADK | adenosine kinase | yes | no | no |  |  |
| NM_001793 | CDH3 | cadherin 3, type 1, P-cadherin (placental) | yes | no | no |  |  |
| NM_004441 | EPHB1 | EphB1 | yes | no | no |  |  |
| NM_024588 | FLJ23584 | hypothetical protein FLJ23584 | yes | no | no |  |  |
| NM_030380 | GLI2 | GLI-Kruppel family member GLI2 isoform alpha | yes | no | yes |  |  |
| NM_030379 | GLI2 | GLI-Kruppel family member GLI2 isoform beta | yes | no | yes |  |  |
| NM_000585 | IL15 | interleukin 15 isoform 1 precursor | yes | no | yes |  |  |
| NM_145332 | MAP3K7 | mitogen-activated protein kinase kinase kinase 7 | yes | no | yes |  |  |
| NM_005115 | MVP | major vault protein | yes | no | no |  |  |
| NM_000620 | NOS1 | nitric oxide synthase 1 (neuronal) | yes | no | no |  |  |
| NM_018206 | ORC6L | origin recognition complex subunit 6 | yes | no | no |  |  |
| NM_006237 | POU4F1 | POU domain, class 4, transcription factor 1 | yes | no | no |  |  |
| NM_006347 | PPIH | peptidyl prolyl isomerase H (cyclophilin H) | yes | no | no |  |  |
| NM_012397 | SERPINB13 | serine (or cysteine) proteinase inhibitor, clade B, member 13 | yes | no | no |  |  |
| NM_005641 | TAF6 | TAF6 RNA polymerase II | yes | no | no |  |  |
| NM_033226 | ABCC12 | ATP-binding cassette, sub-family C (CFTR/MRP), member 12 | no | no | no |  |  |
| NM_004422 | ACADVL | acyl-Coenzyme A dehydrogenase, very long chain | no | no | no |  |  |
| NM_031900 | AGXT2 | alanine-glyoxylate aminotransferase 2 | no | no | no |  |  |
| NM_013449 | AK097342 | Hypothetical protein FLJ40023. | no | no | no |  |  |
| NM_021188 | APA1 | clones 23667 and 23775 zinc finger protein | no | no | no |  |  |
| NM_016442 | ARTS-1 | type 1 tumor necrosis factor receptor shedding aminopeptidase regulator | no | no | no |  |  |
| NM_018489 | ASH1 | hypothetical protein ASH1 | no | no | no |  |  |
| NM_006356 | ATP5H | ATP synthase, H+ transporting, mitochondrial F0 complex, subunit d | no | no | no |  |  |
| NM_000283 | ATP5I | ATP synthase, H+ transporting, mitochondrial F0 | no | no | no |  |  |
| NM_001006117 | AY597808 | Testis-specific Y-linked 6. | no | no | no |  |  |
| NM_001006117 | AY597808 | Testis-specific Y-linked 6. | no | no | no |  |  |
| NM_006020 | C14orf156 | hypothetical protein LOC81892 | no | no | no |  |  |
| NM_020156 | C1GALT1 | core 1 UDP-galactose:N-acetylgalactosamine-alpha-R beta 1,3-galactosyltransferase | no | no | no |  |  |
| NM_015101 | C1orf17 | chromosome 1 open reading frame 17 | no | no | no |  |  |
| NM_030806 | C1orf21 | chromosome 1 open reading frame 21 | no | no | no |  |  |
| NM_031908 | C1QTNF2 | C1q and tumor necrosis factor related protein 2 | no | no | no |  |  |
| NM_018354 | C20orf46 | chromosome 20 open reading frame 46 | no | no | no |  |  |
| NM_000100 | C21orf93 | hypothetical protein LOC246704 | no | no | no |  |  |
| NM_020439 | CAMK1G | calcium/calmodulin-dependent protein kinase IG | no | no | no |  |  |
| NM_003655 | CBX4 | chromobox homolog 4 (Pc class homolog, Drosophila) | no | no | no |  |  |
| NM_016323 | CEB1 | cyclin-E binding protein 1 | no | no | no |  |  |
| NM_006569 | CGR11 | cell growth regulatory with EF-hand domain | no | no | no |  |  |
| NM_001270 | CHD1 | chromodomain helicase DNA binding protein 1 | no | no | no |  |  |
| NM_000742 | CHRNA2 | cholinergic receptor, nicotinic, alpha polypeptide 2 (neuronal) | no | no | no |  |  |
| NM_021101 | CLDN1 | claudin 1 | no | no | yes |  |  |
| NM_001306 | CLDN3 | claudin 3 | no | no | yes |  |  |
| NM_001832 | CLPS | colipase, pancreatic | no | no | no |  |  |
| NM_014515 | CNOT2 | CCR4-NOT transcription complex, subunit 2 | no | no | no |  |  |
| NM_006032 | CPNE6 | copine VI (neuronal) | no | no | no |  |  |
| NM_021181 | CRACC | 19A24 protein | no | no | no |  |  |
| NM_012341 | CRFG | G protein-binding protein CRFG | no | no | no |  |  |
| NM_019604 | CRTAM | class-I MHC-restricted T cell associated molecule | no | no | no |  |  |
| NM_014617 | CRYGA | crystallin, gamma A | no | no | no |  |  |
| NM_001907 | CTRL | chymotrypsin-like | no | no | no |  |  |
| NM_003676 | DEGS | sphingolipid delta 4 desaturase | no | no | no |  |  |
| NM_015644 | DKFZP434B103 | DKFZP434B103 protein | no | no | no |  |  |
| NM_014043 | DKFZP564O123 | DKFZP564O123 protein | no | no | no |  |  |
| NM_004411 | DNCI1 | dynein, cytoplasmic, intermediate polypeptide 1 | no | no | no |  |  |
| NM_021071 | DO | Dombrock blood group | no | no | no |  |  |
| NM_012152 | EDG7 | endothelial differentiation, lysophosphatidic acid G-protein-coupled receptor, 7 | no | no | no |  |  |
| NM_001426 | EN1 | engrailed homolog 1 | no | no | no |  |  |
| NM_198076 | FAM36A | family with sequence similarity 36 member A | no | no | no |  |  |
| NM_017984 | FLJ10057 | hypothetical protein FLJ10057 | no | no | no |  |  |
| NM_018074 | FLJ10374 | hypothetical protein FLJ10374 | no | no | no |  |  |
| NM_018178 | FLJ10687 | hypothetical protein FLJ10687 | no | no | no |  |  |
| NM_018290 | FLJ10983 | hypothetical protein FLJ10983 | no | no | no |  |  |
| NM_024602 | FLJ21156 | hypothetical protein FLJ21156 | no | no | no |  |  |
| NM_000575 | FLJ40629 | hypothetical protein LOC150468 | no | no | no |  |  |
| NM_014745 | FLJ45121 | hypothetical protein LOC400556 | no | no | no |  |  |
| NM_002035 | FVT1 | follicular lymphoma variant translocation 1 | no | no | no |  |  |
| NM_004120 | GBP2 | guanylate binding protein 2, interferon-inducible | no | no | no |  |  |
| NM_018972 | GDAP1 | ganglioside-induced differentiation-associated protein 1 | no | no | no |  |  |
| NM_000557 | GDF5 | growth differentiation factor 5 (cartilage-derived morphogenetic protein-1) | no | no | no |  |  |
| NM_020198 | GK001 | GK001 protein | no | no | no |  |  |
| NM_002065 | GLUL | glutamate-ammonia ligase (glutamine synthase) | no | no | no |  |  |
| NM_030784 | GPR63 | G protein-coupled receptor 63 | no | no | no |  |  |
| NM_016471 | GSDML | hypothetical protein LOC55876 | no | no | no |  |  |
| NM_052851 | GT650 | similar to RhoGAP | no | no | no |  |  |
| NG_000019 | GYS1 | glycogen synthase 1 (muscle) | no | no | no |  |  |
| NM_000182 | HADHB | hydroxyacyl dehydrogenase, subunit B | no | no | no |  |  |
| NM_001523 | HAS1 | hyaluronan synthase 1 | no | no | no |  |  |
| NM_016258 | HGRG8 | high-glucose-regulated protein 8 | no | no | no |  |  |
| NM_021958 | HLX1 | H2.0-like homeo box 1 (Drosophila) | no | no | no |  |  |
| NM_006735 | HOXA2 | homeo box A2 | no | no | no |  |  |
| NM_004502 | HOXB6 | homeo box B6 isoform 1 | no | no | no |  |  |
| NM_002153 | HSD17B2 | hydroxysteroid (17-beta) dehydrogenase 2 | no | no | yes |  |  |
| NM_001538 | HSF4 | heat shock transcription factor 4 | no | no | no |  |  |
| NM_014169 | HSPC134 | HSPC134 protein | no | no | no |  |  |
| NM_016301 | HSU79274 | hypothetical protein LOC29902 | no | no | no |  |  |
| NM_003855 | IL18R1 | interleukin 18 receptor 1 | no | no | no |  |  |
| NM_145805 | ISL2 | ISL2 transcription factor, LIM/homeodomain, (islet-2) | no | no | no |  |  |
| NM_030926 | ITM3 | integral membrane protein 3 | no | no | no |  |  |
| NM_006469 | IVNS1ABP | influenza virus NS1A binding protein isoform b | no | no | no |  |  |
| NM_014673 | KIAA0103 | KIAA0103 gene product | no | no | no |  |  |
| NM_014717 | KIAA0390 | KIAA0390 gene product | no | no | no |  |  |
| NM_012301 | KIAA0705 | atrophin-1 interacting protein 1; activin receptor interacting protein 1 | no | no | no |  |  |
| NM_014513 | KIR2DS5 | killer cell immunoglobulin-like receptor, two domains, short cytoplasmic tail, 5 | no | no | no |  |  |
| NM_002283 | KRTHB5 | keratin, hair, basic, 5 | no | no | no |  |  |
| NM_002284 | KRTHB6 | keratin, hair, basic, 6 (monilethrix) | no | no | no |  |  |
| NM_004522 | LOC130576 | hypothetical protein LOC130576 | no | no | no |  |  |
| NM_019644 | LOC56311 | testis-specific ankyrin motif containing protein | no | no | no |  |  |
| NM_020243 | Tom22 | mitochondrial import receptor Tom22 | no | no | no |  |  |
| NM_004525 | LRP2 | low density lipoprotein-related protein 2 | no | no | no |  |  |
| NM_145161 | MAP2K5 | mitogen-activated protein kinase kinase 5 | no | no | yes |  |  |
| NM_024296 | MGC1203 | hypothetical protein MGC1203 | no | no | no |  |  |
| NM_138290 | MGC26655 | hypothetical protein MGC26655 | no | no | no |  |  |
| NM_004793 | MGC39581 | hypothetical protein LOC257062 | no | no | no |  |  |
| NM_032377 | MGC4549 | hypothetical protein MGC4549 | no | no | no |  |  |
| NM_002416 | MIG | monokine induced by gamma interferon | no | no | no |  |  |
| NM_016640 | MRPS30 | mitochondrial ribosomal protein S30 | no | no | no |  |  |
| NM_000261 | MYOC | myocilin, trabecular meshwork inducible glucocorticoid response | no | no | no |  |  |
| NM_005005 | NDUFB9 | NADH dehydrogenase (ubiquinone) 1 beta subcomplex, 9 (22kD, B22) | no | no | no |  |  |
| NM_002501 | NFIX | Hypothetical protein DKFZp686I05275. | no | no | no |  |  |
| NM_015953 | NOSIP | eNOS interacting protein | no | no | no |  |  |
| NM_002526 | NT5E | 5' nucleotidase, ecto (CD73) | no | no | yes |  |  |
| NM_033282 | OPN4 | opsin 4 (melanopsin) | no | no | no |  |  |
| NM_030903 | OR2W1 | olfactory receptor, family 2, subfamily W, member 1 | no | no | no |  |  |
| NM_020530 | OSM | oncostatin M | no | no | yes |  |  |
| NM_005017 | PCYT1A | phosphate cytidylyltransferase 1, choline, alpha isoform | no | no | no |  |  |
| NM_006211 | PENK | proenkephalin | no | no | yes |  |  |
| NM_002630 | PGC | progastricsin (pepsinogen C) | no | no | yes |  |  |
| NM_017884 | PINX1 | PIN2-interacting protein 1 | no | no | yes |  |  |
| NM_005028 | PIP5K2A | phosphatidylinositol-4-phosphate 5-kinase, type II, alpha | no | no | no |  |  |
| NM_006029 | PNMA1 | paraneoplastic antigen MA1 | no | no | no |  |  |
| NM_005040 | PRCP | prolylcarboxypeptidase isoform 2 | no | no | no |  |  |
| NM_002743 | PRKCSH | protein kinase C substrate 80K-H isoform 2 | no | no | no |  |  |
| NM_018627 | PRO2405 | hypothetical protein PRO2405 | no | no | no |  |  |
| NM_002777 | PRTN3 | proteinase 3 (serine proteinase, neutrophil, Wegener granulomatosis autoantigen) | no | no | no |  |  |
| NM_002780 | PSG4 | pregnancy specific beta-1-glycoprotein 4 isoform | no | no | no |  |  |
| NM_002784 | PSG9 | pregnancy specific beta-1-glycoprotein 9 | no | no | no |  |  |
| NM_002848 | PTPRO | receptor-type protein tyrosine phosphatase O isoform b precursor | no | no | no |  |  |
| NM_080879 | RAB40A | RAB40A, member RAS oncogene family | no | no | no |  |  |
| NM_001798 | RAB5B | RAB5B, member RAS oncogene family | no | no | no |  |  |
| NM_003834 | RGS11 | regulator of G-protein signalling 11 | no | no | no |  |  |
| NM_006480 | RGS14 | regulator of G-protein signalling 14 | no | no | no |  |  |
| NM_000324 | RHAG | Rhesus blood group-associated glycoprotein | no | no | no |  |  |
| NM_021253 | RNF23 | ring finger protein 23 | no | no | no |  |  |
| NM_018119 | RPC5 | RNA polymerase III 80 kDa subunit RPC5 | no | no | no |  |  |
| NM_005619 | RTN2 | reticulon 2 | no | no | no |  |  |
| NM_002730 | SAMD1 | sterile alpha motif domain containing 1 | no | no | no |  |  |
| NM_004636 | SEMA3B | semaphorin 3B isoform 2 precursor | no | no | no |  |  |
| NM_000578 | SLC11A1 | solute carrier family 11 (proton-coupled divalent metal ion transporters), member 1 | no | no | no |  |  |
| NM_005073 | SLC15A1 | solute carrier family 15 (oligopeptide transporter), member 1 | no | no | no |  |  |
| NM_003053 | SLC18A1 | solute carrier family 18 (vesicular monoamine), member 1 | no | no | no |  |  |
| NM_004727 | SLC24A1 | solute carrier family 24 (sodium/potassium/calcium exchanger), member 1 | no | no | no |  |  |
| NM_016180 | SLC45A2 | solute carrier family 45 | no | no | no |  |  |
| NM_003044 | SLC6A12 | solute carrier family 6 (neurotransmitter transporter, betaine/GABA), member 12 | no | no | no |  |  |
| NM_003043 | SLC6A6 | solute carrier family 6 (neurotransmitter transporter, taurine), member 6 | no | no | no |  |  |
| NM_003063 | SLN | sarcolipin | no | no | no |  |  |
| NM_031953 | SNX25 | sorting nexin 25 | no | no | no |  |  |
| NM_003108 | SOX11 | SRY (sex determining region Y)-box 11 | no | no | no |  |  |
| NM_005274 | SPATA1 | spermatogenesis associated 1 | no | no | no |  |  |
| NM_015493 | SPBC24 | spindle pole body component 24 homolog | no | no | no |  |  |
| NM_003127 | SPTAN1 | SPTAN1 protein (Fragment). | no | no | no |  |  |
| NM_003155 | STC1 | stanniocalcin 1 | no | no | no |  |  |
| NM_007269 | STXBP3 | syntaxin binding protein 3 | no | no | no |  |  |
| NM_004607 | TBCA | tubulin-specific chaperone a | no | no | no |  |  |
| NM_007005 | TLE4 | BCE-1 protein. | no | no | no |  |  |
| NM_032871 | TNFRSF19L | tumor necrosis factor receptor superfamily, | no | no | no |  |  |
| NM_000074 | TNFSF5 | tumor necrosis factor (ligand) superfamily, member 5 | no | no | no |  |  |
| NM_005425 | TNP2 | transition protein 2 (during histone to protamine replacement) | no | no | no |  |  |
| NM_007218 | TRC8 | patched related protein translocated in renal cancer | no | no | no |  |  |
| NM_019841 | TRPV5 | transient receptor potential cation channel, subfamily V, member 5 | no | no | no |  |  |
| NM_016032 | ZDHHC9 | zinc finger, DHHC domain containing 9 | no | no | no |  |  |
| NM_003457 | ZNF207 | zinc finger protein 207 | no | no | no |  |  |
| NM_003419 | ZNF345 | zinc finger protein 345 | no | no | no |  |  |
| NM_007145 | ZNF565 | zinc finger protein 565 | no | no | no |  |  |
| NM_003426 | ZNF74 | zinc finger protein 74 (Cos52) | no | no | no |  |  |
| NM_018525 | AF119879 | PRO2369. | no | no | no |  |  |
| NM_024690 | AK024365 | Hypothetical protein FLJ14303. | no | no | no |  |  |
| NM_002847 | AK127222 | Hypothetical protein FLJ45289. | no | no | no |  |  |
| NM_012404 | ANP32D | acidic (leucine-rich) nuclear phosphoprotein 32 family, member D | no | no | no |  |  |
| NM_004707 | AP3S1 | adaptor-related protein complex 3, sigma 1 | no | no | no |  |  |
| NM_005503 | APBA2 | amyloid beta (A4) precursor protein-binding, family A, member 2 (X11-like) | no | no | no |  |  |
| NM_030643 | APOL4 | apolipoprotein L, 4 | no | no | no |  |  |
| NM_007044 | BC071678 | Hypothetical protein. | no | no | no |  |  |
| NM_003666 | BLZF1 | basic leucine zipper nuclear factor 1 (JEM-1) | no | no | no |  |  |
| NM_012108 | BRDG1 | BCR downstream signaling 1 | no | no | no |  |  |
| NM_000491 | C1QB | complement component 1, q subcomponent, beta polypeptide | no | no | no |  |  |
| NM_005750 | C4orf6 | chromosome 4 open reading frame 6 | no | no | no |  |  |
| NM_000255 | C6orf139 | hypothetical protein LOC55166 | no | no | no |  |  |
| NM_030893 | CD1E | CD1E antigen, e polypeptide | no | no | no |  |  |
| NM_001836 | CMA1 | chymase 1, mast cell | no | no | no |  |  |
| NM_001888 | CRYM | crystallin, mu | no | no | yes |  |  |
| NM_000759 | CSF3 | colony stimulating factor 3 (granulocyte) | no | no | no |  |  |
| NM_021187 | CYP4F11 | cytochrome P450, subfamily IVF, polypeptide 11 | no | no | no |  |  |
| NM_016221 | DCTN4 | Homo sapiens dynactin 4 (p62) (DCTN4), mRNA. | no | no | no |  |  |
| NM_015544 | DKFZP564K1964 | DKFZP564K1964 protein | no | no | no |  |  |
| NM_030953 | DKFZP761E2110 | hypothetical protein DKFZp761E2110 | no | no | no |  |  |
| NM_021951 | DMRT1 | doublesex and mab-3 related transcription factor 1 | no | no | no |  |  |
| NM_022552 | DNMT3A | DNA cytosine methyltransferase 3 alpha isoform | no | no | yes |  |  |
| NM_000137 | FAH | fumarylacetoacetate hydrolase (fumarylacetoacetase) | no | no | no |  |  |
| NM_018208 | FLJ10761 | hypothetical protein FLJ10761 | no | no | no |  |  |
| NM_019028 | FLJ10852 | hypothetical protein similar to ankyrin repeat-containing priotein AKR1 | no | no | no |  |  |
| NM_025188 | FLJ13181 | hypothetical protein FLJ13181 | no | no | no |  |  |
| NM_024521 | FLJ21459 | hypothetical protein FLJ21459 | no | no | no |  |  |
| NM_021024 | FLJ23588 | CAP-binding protein complex interacting protein | no | no | no |  |  |
| NM_021039 | FLJ36166 | hypothetical protein LOC349152 | no | no | no |  |  |
| NM_014785 | GBA2 | bile acid beta-glucosidase | no | no | no |  |  |
| NM_004837 | GGPS1 | geranylgeranyl diphosphate synthase 1 | no | no | no |  |  |
| NM_005297 | GPR24 | G protein-coupled receptor 24 | no | no | no |  |  |
| NM_032551 | GPR54 | G protein-coupled receptor 54 | no | no | yes |  |  |
| NM_005285 | GPR7 | G protein-coupled receptor 7 | no | no | yes |  |  |
| NM_014707 | HDAC9-PENDING | histone deacetylase 9 | no | no | no |  |  |
| NM_000200 | HTN3 | histatin 3 | no | no | no |  |  |
| NM_014216 | ITPK1 | Homo sapiens inositol 1,3,4-triphosphate 5/6 kinase (ITPK1) | no | no | no |  |  |
| NM_014217 | KCNK2 | potassium channel, subfamily K, member 2 (TREK-1) | no | no | no |  |  |
| NM_002246 | KCNK3 | potassium channel, subfamily K, member 3 (TASK-1) | no | no | no |  |  |
| NM_003685 | KHSRP | KH-type splicing regulatory protein (FUSE binding protein 2) | no | no | no |  |  |
| NM_020535 | KIR2DL2 | killer cell immunoglobulin-like receptor, two | no | no | no |  |  |
| NG_000919 | KRTAP12-1 | keratin associated protein 12-1 | no | no | no |  |  |
| NM_012317 | LDOC1 | leucine zipper, down-regulated in cancer 1 | no | no | yes |  |  |
| NM_016735 | LIMK1 | LIM domain kinase 1 isoform 1 | no | no | yes |  |  |
| NM_032372 | LOC143241 | BC019250 protein | no | no | no |  |  |
| NM_004811 | LPXN | leupaxin | no | no | no |  |  |
| NM_003980 | MAP7 | microtubule-associated protein 7 | no | no | no |  |  |
| NM_031466 | MGC4737 | KIAA1882 protein | no | no | no |  |  |
| NM_002451 | MTAP | methylthioadenosine phosphorylase | no | no | yes |  |  |
| NG_001007 | MTCO2L | cytochrome c oxidase II-like | no | no | no |  |  |
| NM_032623 | OSAP | ovary-specific acidic protein | no | no | no |  |  |
| NM_052959 | PANX3 | pannexin 3 | no | no | no |  |  |
| NM_005044 | PRKX | protein kinase, X-linked | no | no | no |  |  |
| NM_006905 | PSG1 | pregnancy specific beta-1-glycoprotein 1 | no | no | no |  |  |
| NM_005608 | PTPRCAP | protein tyrosine phosphatase, receptor type, C-associated protein | no | no | no |  |  |
| NM_017790 | RGS3 | regulator of G-protein signalling 3 isoform 5 | no | no | no |  |  |
| NM_014554 | SENP1 | SUMO1/sentrin specific protease 1 | no | no | yes |  |  |
| NM_000337 | SGCD | sarcoglycan, delta (35kD dystrophin-associated glycoprotein) | no | no | no |  |  |
| NM_021097 | SLC8A1 | solute carrier family 8 (sodium/calcium exchanger), member 1 | no | no | no |  |  |
| NM_003104 | SORD | sorbitol dehydrogenase | no | no | no |  |  |
| NM_007173 | SPUVE | protease, serine, 23 | no | no | no |  |  |
| NG_001058 | ST13P | suppression of tumorigenicity 13 (colon carcinoma) (Hsp70 interacting protein) pseudogene | no | no | no |  |  |
| NM_004800 | TM9SF2 | transmembrane 9 superfamily member 2 | no | no | no |  |  |
| NM_004620 | TRAF6 | TNF receptor-associated factor 6 | no | no | no |  |  |
| NM_014232 | VAMP2 | vesicle-associated membrane protein 2 (synaptobrevin 2) | no | no | no |  |  |
