# Supplementary material for: Egr1 regulates the coordinated expression of numerous EGF receptor target genes as identified by ChIP-on-chip
Source: Genome Biol. 2008 Nov 25;9(11):R166. doi: 10.1186/gb-2008-9-11-r166 (PMC2614498; doi:10.1186/gb-2008-9-11-r166)
Supplement: Additional data file 3 — Table S2: log fold changes and p-values of the Egr1 target genes that were significantly differentially expressed between UV treated and mock cells using Affymetrix HGU133plus 2 arrays. Table S3: the gene expression analysis of Egr1 target genes using qRT-PCR of RNA extracted at various time points after UV-C treatment of DU145 cells. [file gb-2008-9-11-r166-S3.doc]

**Supplemental Table S2:** Egr1 target genes that were significantly differentially expressed between UV treated and mock cells using Affymetrix HGU133plus 2 arrays. FC: Fold Change

| Gene Symbol | Log FC | p value |
| --- | --- | --- |
| ALS2CR2 | 1.177 | 0.001 |
| ARHGEF12 | 0.389 | 0.049 |
| B4GALT1 | 1.129 | 0.005 |
| CBX4 | 0.806 | 0.004 |
| CD109 | -0.580 | 0.018 |
| CDK8 | -0.449 | 0.027 |
| CHD11 | -1.411 | 0.006 |
| CLDN1 | -0.464 | 0.034 |
| CNOT2 | -0.402 | 0.040 |
| DYRK2 | -0.833 | 0.043 |
| EIF2 | -1.559 | 0.000 |
| ELF2 | -1.492 | 0.011 |
| FOSL2 | -0.614 | 0.011 |
| GEMIN4 | 0.441 | 0.043 |
| H3AF | 1.084 | 0.009 |
| HNRPD | -2.830 | 0.002 |
| IGFBP6 | 0.380 | 0.042 |
| KLF12 | -0.845 | 0.008 |
| KLF7 | -1.015 | 0.003 |
| MAP2K5 | -0.385 | 0.033 |
| MAP3K7IP3 | -0.439 | 0.041 |
| NOSIP | 0.373 | 0.039 |
| PSMB10 | 0.410 | 0.046 |
| RASSF5 | -0.503 | 0.023 |
| RGS14 | 0.450 | 0.047 |
| SLC6A6 | -0.773 | 0.011 |
| SPBC24 | 0.396 | 0.043 |
| SPTAN1 | 0.907 | 0.002 |
| TBCA | 1.187 | 0.001 |
| TLE4 | -0.969 | 0.003 |
| ZNF 565 | -0.763 | 0.004 |
| ZNF207 | -1.914 | 0.000 |

**Supplemental Table S3:** Gene expression analysis of Egr1 target genes using qRT-PCR of RNA extracted at various time points after UV-C treatment of DU145 cells

|  | - Control | - 30' | - 1h | - 2h | - 6h | - 16h |
| --- | --- | --- | --- | --- | --- | --- |
| - ABCC3 | - 1 | - 0.76 | - 0.95 | - 0.84 | - 0.84 | - 0.74 |
| - AKAP9 | - 1 | - 0.62 | - 0.37 | - 0.58 | - 0.47 | - 0.34 |
| - BBC3 | - 1 | - 1.20 | - 21.21 | - 4.27 | - 0.58 | - 0.05 |
| - BLK | - 1 | - 1.54 | - 1.02 | - 0.88 | - 1.05 | - 1.79 |
| - BMP4 | - 1 | - 0.51 | - 0.57 | - 0.53 | - 0.11 | - 0.04 |
| - CASP7 | - 1 | - 0.76 | - 0.78 | - 0.64 | - 0.41 | - 0.23 |
| - CDK8 | - 1 | - 0.81 | - 1.04 | - 1.07 | - 0.53 | - 0.29 |
| - CITED4 | - 1 | - 0.98 | - 0.99 | - 0.89 | - 0.54 | - 0.23 |
| - CysLTR1 | - 1 | - 0.72 | - 1.69 | - 2.68 | - 1.44 | - 2.50 |
| - DYRK2 | - 1 | - 0.54 | - 0.44 | - 0.39 | - 0.08 | - 0.05 |
| - EGFR | - 1 | - 0.51 | - 0.47 | - 0.71 | - 0.37 | - 0.18 |
| - Egr2 | - 1 | - 0.81 | - 2.33 | - 3.00 | - 0.42 | - 0.61 |
| - ELF2 | - 1 | - 0.78 | - 0.61 | - 0.54 | - 0.13 | - 0.06 |
| - ETS2 | - 1 | - 0.46 | - 0.56 | - 0.59 | - 0.19 | - 0.21 |
| - FosL2 | - 1 | - 0.64 | - 0.68 | - 0.49 | - 0.25 | - 0.15 |
| - GMPS1 | - 1 | - 0.90 | - 0.54 | - 0.79 | - 0.57 | - 0.39 |
| - GSTA3 | - 1 | - 0.95 | - 0.46 | - 0.80 | - 0.85 | - 0.73 |
| - H3F3A | - 1 | - 0.85 | - 0.62 | - 0.67 | - 0.69 | - 0.56 |
| - HK1 | - 1 | - 1.25 | - 1.72 | - 0.48 | - 0.76 | - 0.32 |
| - HNRPDP | - 1 | - 0.85 | - 0.88 | - 1.01 | - 0.47 | - 0.78 |
| - IFITM2 | - 1 | - 1.05 | - 1.13 | - 1.17 | - 0.86 | - 0.80 |
| - IL11RA | - 1 | - 0.27 | - 0.32 | - 0.47 | - 0.40 | - 0.39 |
| - MAP2 | - 1 | - 0.50 | - 0.35 | - 0.44 | - 0.33 | - 0.27 |
| - MAP4 | - 1 | - 0.78 | - 0.76 | - 0.47 | - 0.55 | - 0.56 |
| - MAX | - 1 | - 1.03 | - 0.64 | - 0.64 | - 0.00 | - 0.25 |
| - NME1 | - 1 | - 0.91 | - 1.00 | - 0.98 | - 1.00 | - 0.93 |
| - NME2 | - 1 | - 1.07 | - 1.01 | - 1.97 | - 1.53 | - 0.95 |
| - PAK6 | - 1 | - 0.62 | - 0.75 | - 0.80 | - 0.41 | - 0.24 |
| - PSD3 | - 1 | - 0.97 | - 0.78 | - 1.02 | - 0.79 | - 0.94 |
| - PTPN13 | - 1 | - 0.49 | - 0.64 | - 0.57 | - 0.41 | - 0.43 |
| - PTPRO | - 1 | - 0.46 | - 0.68 | - 0.71 | - 1.33 | - 2.25 |
| - RRAS2 | - 1 | - 0.90 | - 0.70 | - 0.80 | - 0.53 | - 0.76 |
| - SLP1 | - 1 | - 1.14 | - 1.02 | - 0.74 | - 0.71 | - 0.60 |
| - TNFSF6 | - 1 | - 0.83 | - 0.21 | - 1.66 | - 1.45 | - 0.84 |
| - TOM22 | - 1 | - 0.93 | - 0.81 | - 0.87 | - 0.92 | - 0.77 |
