# Supplementary material for: Egr1 regulates the coordinated expression of numerous EGF receptor target genes as identified by ChIP-on-chip
Source: Genome Biol. 2008 Nov 25;9(11):R166. doi: 10.1186/gb-2008-9-11-r166 (PMC2614498; doi:10.1186/gb-2008-9-11-r166)
Supplement: Additional data file 4 — Figure S1: diagram showing previously reported interactions between EGFR and 23 other Egr1 target genes using Pathway Studio (Ariadne Inc.). Figure S2: plot showing the distribution of fold change values from Affymetrix gene expression data and M values from ChIP-on-chip data for Egr1 target genes. Figure S3: diagram showing previously reported interactions between Egr1's target genes using Pathway Studio (Ariadne Inc.). Figure S4: gene expression of p53, p73, TGFb1 and PTEN was studied by qRT-PCR analysis of RNA isolated from M12 cells at various time points after UV-C treatment. [file gb-2008-9-11-r166-S4.ppt]

## Slide 1
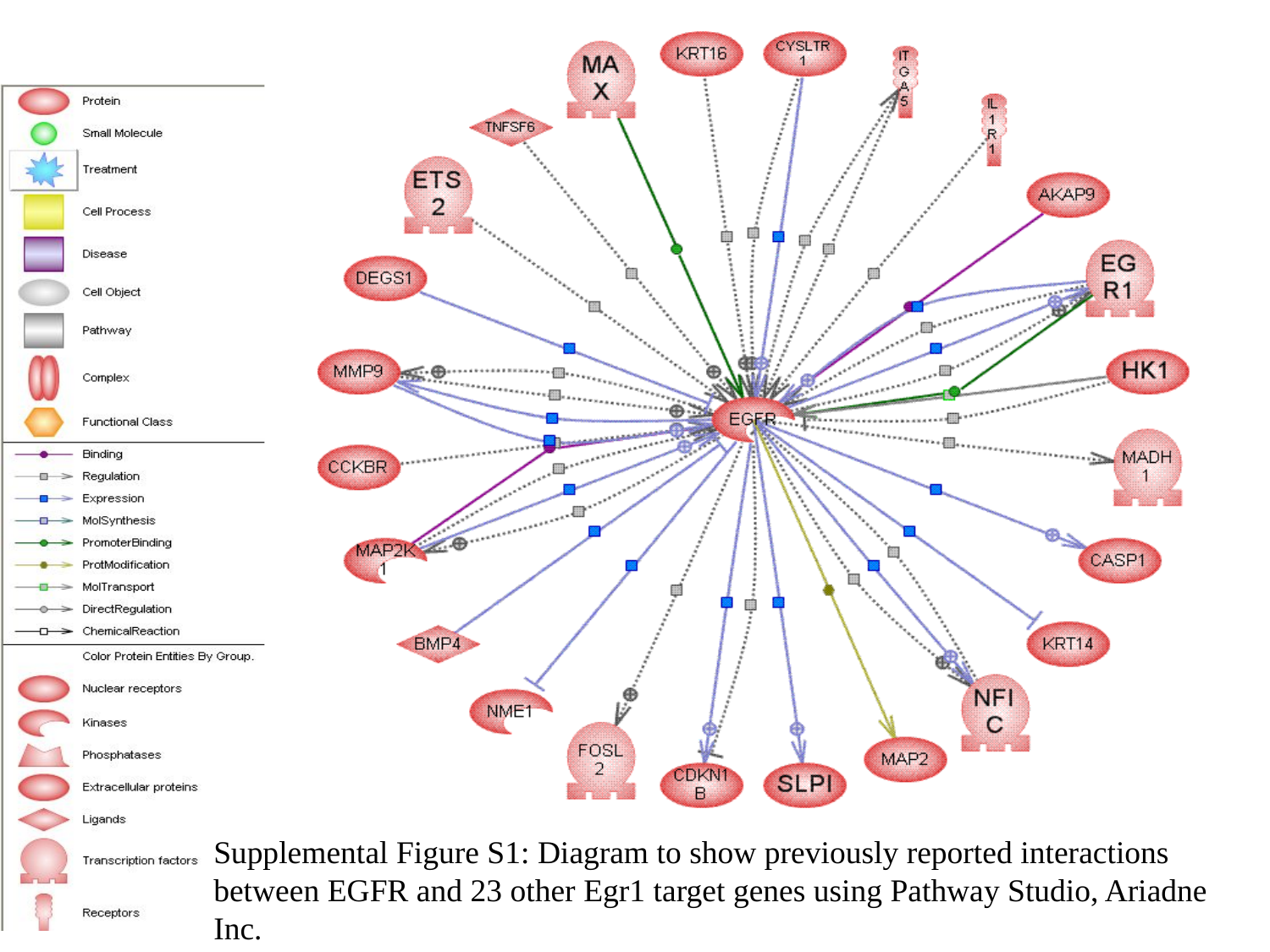

Supplemental Figure S1: Diagram to show previously reported interactions between EGFR and 23 other Egr1 target genes using Pathway Studio, Ariadne Inc.

## Slide 2
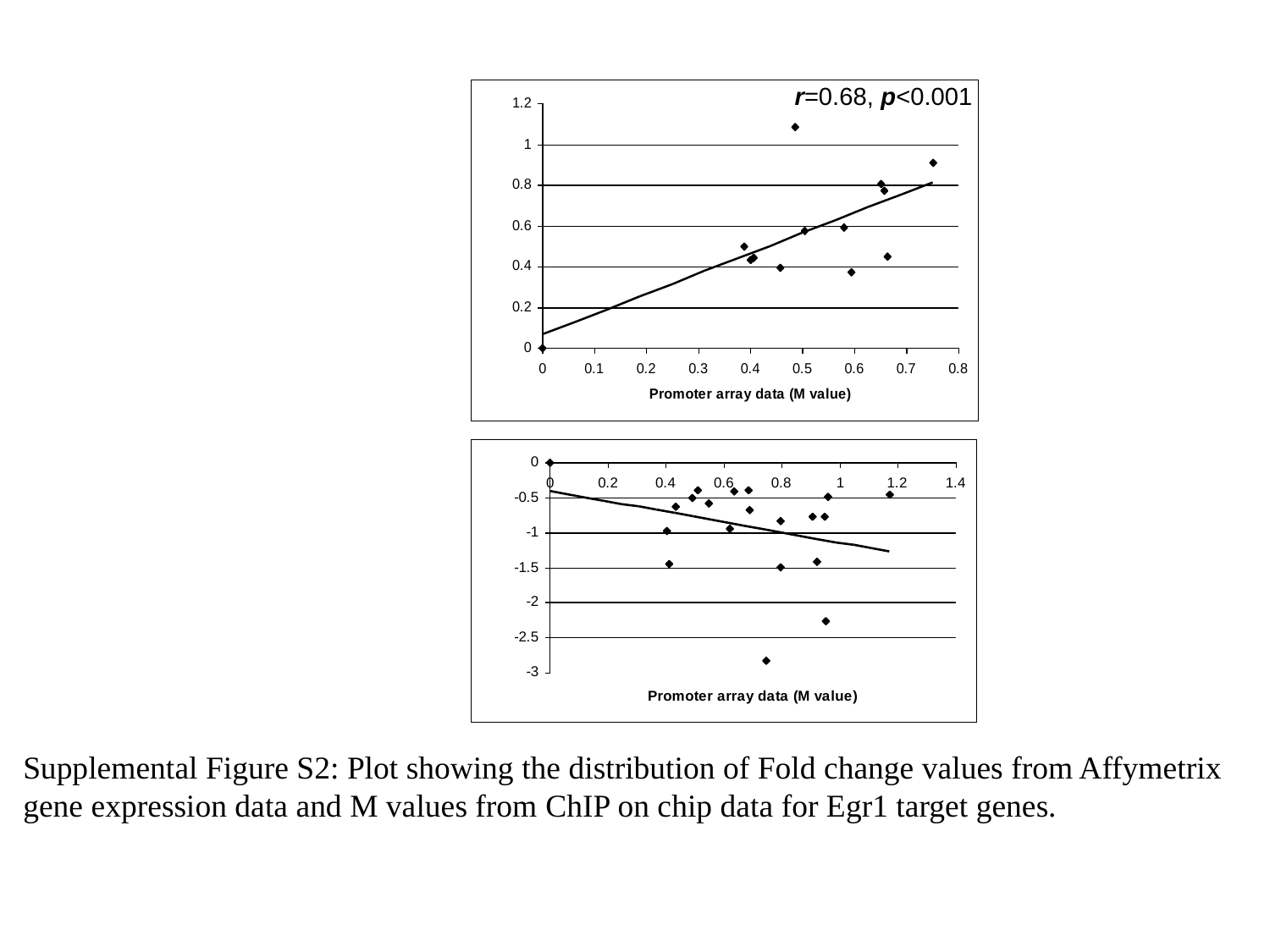

r=0.68, p<0.001
Supplemental Figure S2: Plot showing the distribution of Fold change values from Affymetrix gene expression data and M values from ChIP on chip data for Egr1 target genes.

## Slide 3
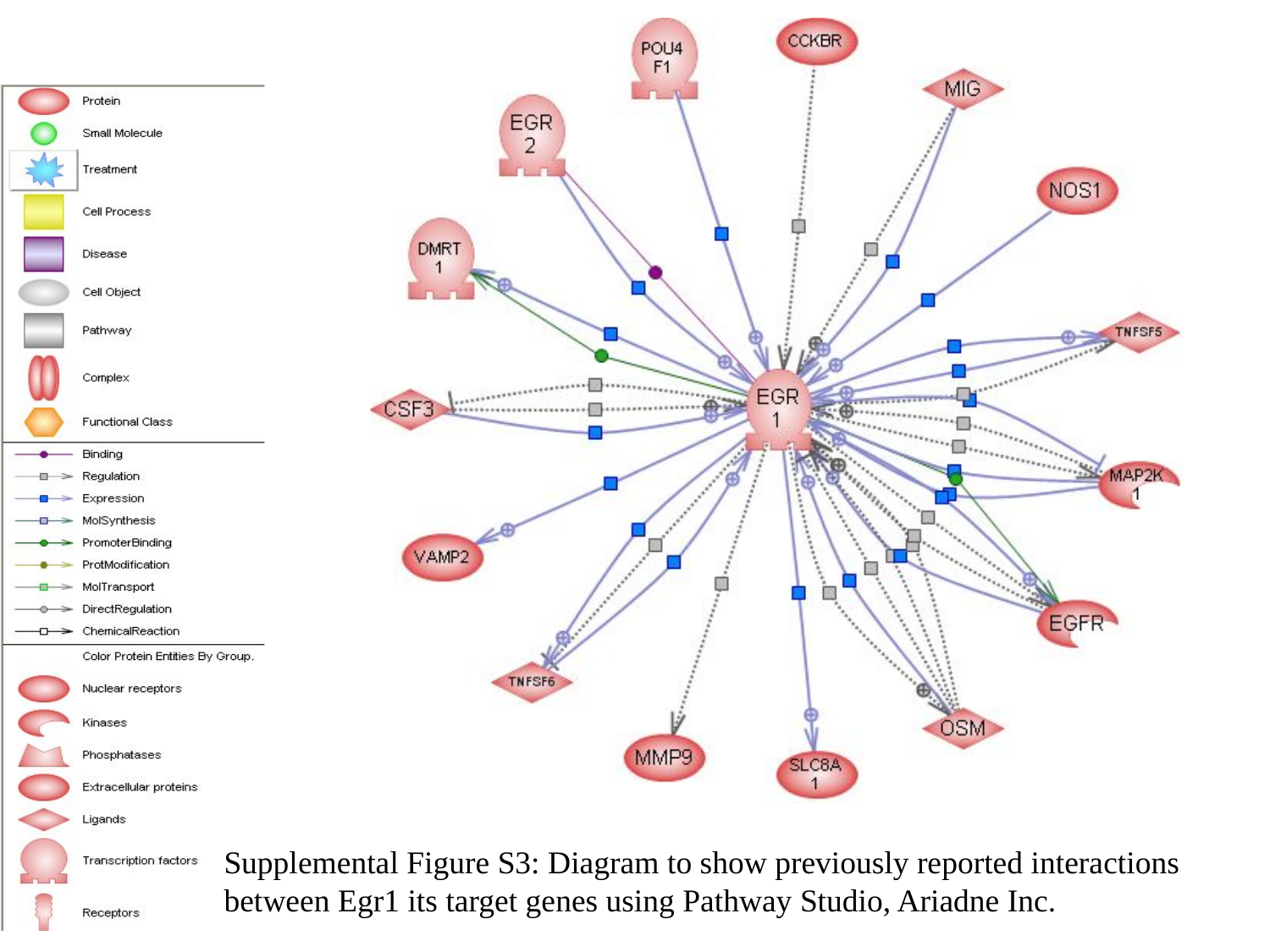

Supplemental Figure S3: Diagram to show previously reported interactions between Egr1 its target genes using Pathway Studio, Ariadne Inc.

## Slide 4
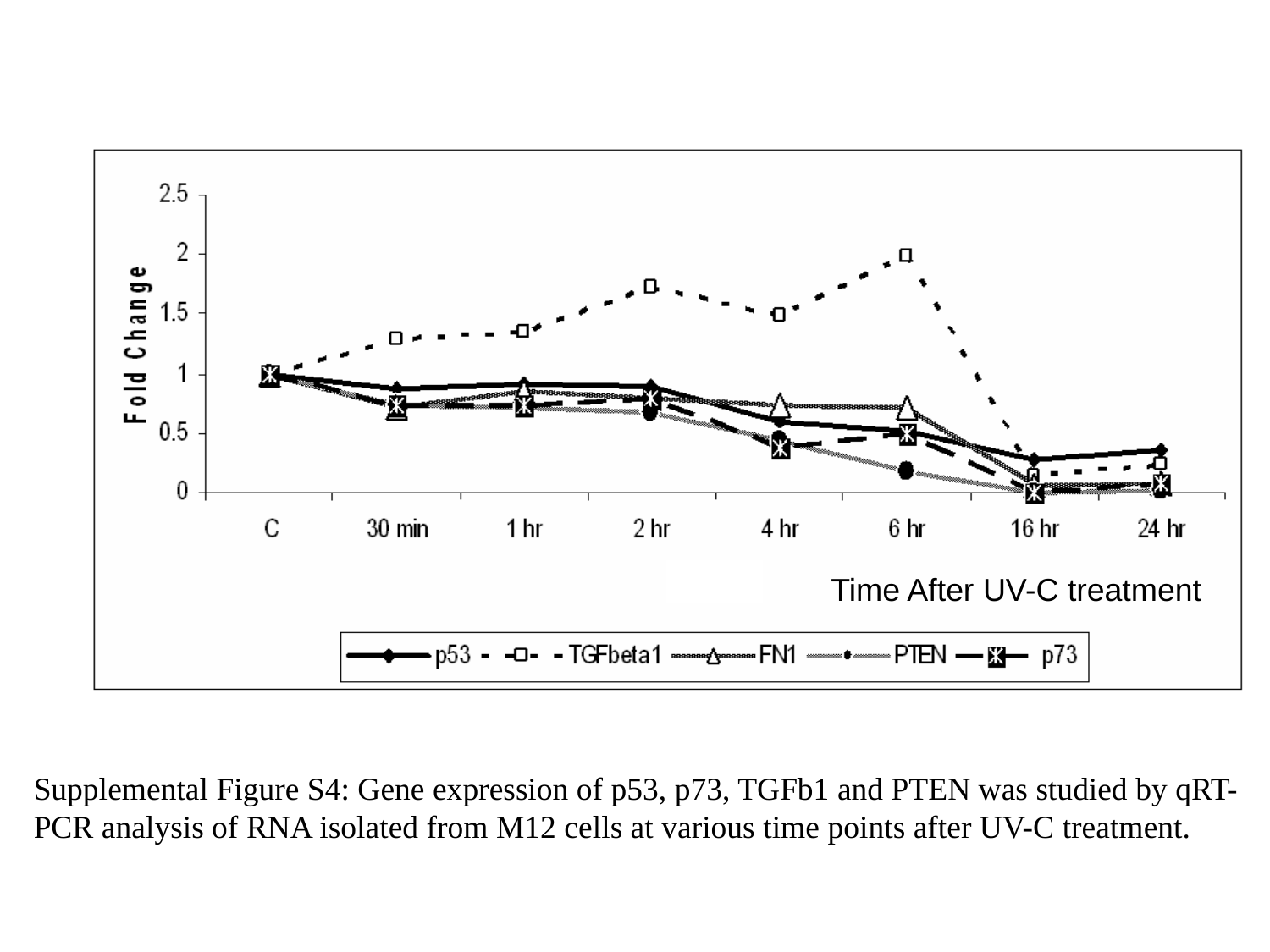

Time After UV-C treatment
Supplemental Figure S4: Gene expression of p53, p73, TGFb1 and PTEN was studied by qRT-PCR analysis of RNA isolated from M12 cells at various time points after UV-C treatment.
